# Language in job advertisements and the reproduction of labor force gender and racial segregation

**DOI:** 10.1093/pnasnexus/pgae526

**Published:** 2024-11-20

**Authors:** Yang Hu, Nicole Denier, Lei Ding, Monideepa Tarafdar, Alla Konnikov, Karen D Hughes, Shenggang Hu, Bran Knowles, Enze Shi, Jabir Alshehabi Al-Ani, Irina Rets, Linglong Kong, Dengdeng Yu, Hongsheng Dai, Bei Jiang

**Affiliations:** Department of Sociology, Lancaster University, Lancaster LA1 4YN, United Kingdom; Department of Sociology, University of Alberta, Edmonton, AB T6G 2H4, Canada; Department of Mathematical and Statistical Sciences, University of Alberta, Edmonton, AB T6G 2J5, Canada; Isenberg School of Management, University of Massachusetts Amherst, Amherst, MA 01003, USA; Department of Management Science, Lancaster University, Lancaster LA1 4YX, United Kingdom; Department of Social Sciences, Concordia University of Edmonton, Edmonton, AB T5B 4E4, Canada; Department of Sociology, University of Alberta, Edmonton, AB T6G 2H4, Canada; Department of Strategy, Entrepreneurship, and Management, University of Alberta, Edmonton, AB T6G 2R6, Canada; Diana International Research Institute, Babson College, Wellesley, MA 02481, USA; Department of Statistics, University of Warwick, Coventry CV4 7AL, United Kingdom; School of Computing and Communications, Lancaster University, Lancaster LA1 4WA, United Kingdom; Department of Mathematical and Statistical Sciences, University of Alberta, Edmonton, AB T6G 2J5, Canada; Department of Computer Science and Data Science, York St. John University, York YO31 7EX, United Kingdom; Department of Management Science, Lancaster University, Lancaster LA1 4YX, United Kingdom; Institute of Educational Technology, The Open University, Milton Keynes MK7 6AA, United Kingdom; Department of Mathematical and Statistical Sciences, University of Alberta, Edmonton, AB T6G 2J5, Canada; Department of Management Science and Statistics, Alvarez College of Business, University of Texas at San Antonio, San Antonio, TX 78249, USA; School of Mathematics, Statistics and Physics, Newcastle University, Newcastle upon Tyne NE1 7RU, United Kingdom; Department of Mathematical and Statistical Sciences, University of Alberta, Edmonton, AB T6G 2J5, Canada

**Keywords:** gender, job advertisement, labor market, race, segregation

## Abstract

Job advertisements (ads) represent the first point of contact between employers and job seekers. By signaling characteristics expected of an ideal candidate, job ads “gatekeep” the labor force and configure its composition. Meanwhile, labor force composition can also shape the wording of job ads. This study develops a multidimensional inventory of gender and EDI (equality, diversity, inclusion) language in job ads. Applying this inventory, it adopts an instrumental-variable approach to disentangle the reciprocal relationships between gender/EDI language in job ads and labor force gender/racial composition. Drawing on the analysis of 28.6 million job ads in the United Kingdom in combination with labor force statistics between 2018 and 2023, the findings reveal three distinct mechanisms through which the bidirectional interplay between language in job ads and labor force composition (re)produces or disrupts labor force gender/racial segregation. They highlight both the benefits and limitations of intervening in the language used in job ads to help reduce labor force gender/racial segregation.

**Significance Statement**

Gender and racial segregation represent persistent and key forms of inequality in the labor market, and job advertisements (ads) “gatekeep” the labor force as the first point of contact between job seekers and employers. Analyzing 28.6 million job ads and labor force statistics, our labor-market-wide auditing study reveals distinct ways in which the bidirectional interplay between gender/EDI language in job ads and labor force composition (re)produces or disrupts labor force gender/racial segregation.

## Introduction

EDI (equality, diversity, inclusion) is increasingly mainstreamed into labor standards, management and organizational practices, and legislation (1). Much as employers, human resource (HR) professionals, job advertising platforms, and policymakers strive to enhance EDI at work, persistent labor force segregation along the lines of gender and race poses a major challenge to achieving EDI in organizations and across labor markets (2, 3). Labor force gender and racial segregation not only represent prominent forms of workplace and labor market inequality, they are also key drivers of gender and racial disparities in income, job satisfaction, and worker well-being (2, 4, 5). Research shows that employees working in more diverse and inclusive organizations are more loyal and better motivated, hence more productive (6, 7). Therefore, it is not surprising that intense research, policy, and management efforts are devoted to reducing gender and racial segregation in order to enhance EDI and bolster productivity in the labor force (1, 7, 8).

Among the many areas of EDI intervention, job advertisements (ads) have garnered growing attention (9–19). Job ads constitute the first point of contact between employers and job seekers, thus playing a crucial role in “gatekeeping” the labor force. Job ads signal explicit and implicit characteristics expected of an ideal candidate (12, 13). Such characteristics—conveyed through particular ways in which job ads are worded—are closely embedded in broader, and often gendered and racialized, social structures that shape both language use and labor market configurations (3, 19). On the labor demand side, job ads are carefully worded to reflect employers’ identities and aspirations, and HR professionals draw on characteristics stated in job ads to formulate criteria for shortlisting and interviewing applicants (17, 20). On the labor supply side, job seekers self-assess their suitability for a job based on those characteristics. For example, psychological experiments show that women perceive jobs to be less appealing or suitable when job ads include a large number of masculine words, such as “active” and “decisive” (11, 12, 19). Similarly, experiments show that racial minority individuals are deterred by job ads lacking racial diversity or containing phrases associated with negative racial stereotypes in their language (15, 21, 22). Consequently, language in job ads can differentially affect job seekers’ inclination to apply for a job across different social groups (12, 15, 19).

Against this backdrop and as part of broader social, political, and legislative shifts toward the use of nondiscriminatory and inclusive language, extensive efforts have been made to diversify and debias language in job ads, in the hope that such efforts may help enhance EDI and reduce gender and racial segregation in the labor market (1, 9, 10, 14, 15). Although both demand-side and supply-side mechanisms suggest that language in job ads can causally impact labor force gender/racial composition, such impact is yet to be substantiated by labor-market-wide audits, beyond individual-level experiments (11, 12, 15). As a result, little is known about the effectiveness of interventions in how job ads are worded in tackling labor force gender and racial segregation. Addressing this substantive gap, our *first* objective is to provide large-scale auditing evidence on the impact of gender/EDI language in job ads on labor force gender/racial composition.

Whereas research has focused predominantly on the impact of job ads on individual job seekers (10–13, 15, 19), far less is known about how labor force composition shapes the wording of job ads. Addressing this knowledge gap will shed light on the production of job ads and provide insights that are crucial to mitigating the impact of job ads on labor force composition. It will also bring to light potential reciprocal relationships between language in job ads and labor force composition, which is a key to developing a systematic, comprehensive understanding of how the interplay between job ads and labor force composition (re)produces or disrupts labor force gender and racial segregation. Our *second* objective, therefore, is to examine the impact of labor force gender/racial composition on gender/EDI language in job ads.

**Fig. 1.**
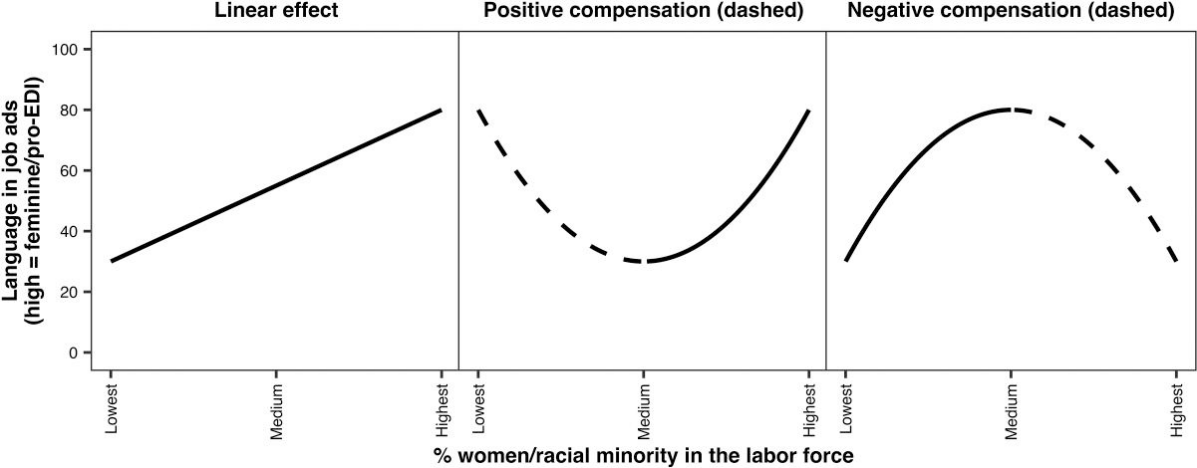
Three hypothetical scenarios of the impact of labor force gender/racial composition on gender/EDI language in job ads. Dashed stretches of the curves indicate the compensation hypotheses.

Specifically, as depicted in Fig. 1, we hypothesize three scenarios of how labor force composition shapes language in job ads. On the one hand, identity theory posits that people's identities are reflected in their language (23)—a tendency that is formulated through long-term socialization and regulated by sociocultural norms as to what constitutes “appropriate” language, for example, for women and men. As job ads emerge from the collective majority identity of a workforce, how the ads are worded may reflect the predominant traits that characterize the workforce's composition. If so, hypothesis 1 predicts that job ads for workforces with a larger share of women as opposed to men include more words (and phrases) that are socially constructed and understood to denote a feminine rather than a masculine orientation; and those for workforces with a larger share of women and racial minority workers may include more EDI words (“linear effect” in Fig. 1).

On the other hand, employers are faced with mounting cultural and political pressure and a legal imperative to enhance EDI (1, 9). Movements, such as #MeToo and #BlackLivesMatter, have re-centered attention on gender and racial segregation as key barriers to achieving EDI in the labor market (24, 25). In response to these recent developments, employers may take a reflective approach to writing job ads and make conscious efforts to strategically word job ads to rectify a lack of gender/racial diversity in the workforce through a “compensation” mechanism (9, 26), which could take two distinct forms. Hypothesis 2 (“positive compensation” in Fig. 1) posits that employers play up language associated with underrepresented groups in the workforce and EDI in job ads. Conversely, hypothesis 3 (“negative compensation” in Fig. 1) posits that employers suppress language associated with majority groups in the workforce. For the first time, our study tests these mechanisms.

Based on the above discussion, our study examines the reciprocal relationships between gender/EDI language in job ads and labor force gender/racial composition. To do so, we developed a new, theoretically informed word inventory that systematically captures six distinct dimensions of gender/EDI language in job ads. Based on this inventory, we used natural language processing and, specifically, word embeddings—a technique that is increasingly used in the latest research on job ads—to comprehensively quantify each dimension of gender/EDI language in the job ads we examined (16–19). We further adopted an instrumental-variable (IV) modeling approach to disentangle the bidirectional influences between language in job ads and labor force composition (27). Providing large-scale labor-market-wide evidence, our analysis draws on 28.6 million job ads, combined with data on the gender and racial composition of the labor force, between 2018 and 2023 in the United Kingdom (see SI Appendix, Supplementary Material 1 for a discussion of the UK labor market context). Although our empirical materials focus on the United Kingdom, we expect our substantive insights, empirical approach, and findings to enjoy broader relevance in other contexts that face similar challenges of labor market gender and racial segregation and are undergoing similar EDI movements.

## Results

### Measuring labor force gender/racial composition and gender/EDI language in job ads

To capture labor force gender and racial composition, we used the UK Quarterly Labor Force Survey (LFS) between January 2018 and June 2023 (*n* = 782,189 working respondents). Specifically, as the gender/racial composition of the same occupation (e.g. managers) varies across different industries (e.g. education vs. manufacturing) (28), we positioned occupations in their industrial settings by creating 189 industry-occupation groups based on the cross-tabulation between the first levels of the Standard Industry Classification 2007 (SIC1) and Standard Occupation Classification 2010 (SOC1). We calculated the percentages of women and non-White racial minority workers across the 189 groups to measure labor force gender/racial composition; we used the LFS weights to ensure our measures are representative of the UK working population. See SI Appendix, Supplementary Material 2 for detailed information on and descriptive statistics for the labor force composition measures. Although we used the proportion of non-White workers to measure labor force racial composition, all our results are robust to using the Blau diversity index. This index captures the probability that two randomly selected individuals from an industry-occupation group belong to two different ethnic groups, which was calculated based on multiple racial/ethnic groups (see SI Appendix, Supplementary Material 8, Table S14).

We developed a six-dimensional word inventory to systematically measure gender/EDI language in job ads, as illustrated below (see SI Appendix, Supplementary Material 3 for the full inventory and information on the inventory's theoretical bases, development, and validation):

1. Building on linguistic research (29), *explicit gender references* include gendered (pro)nouns, such as “she/he,” “his/her,” and “woman/man,” which explicitly signal the gender orientation of a job ad.
2. *Gendered psychological cues* expand on the Bems’ and Gaucher et al.'s word inventories (11, 12, 29). Such cues include words associated with normative gender orientations. For example, communal attributes such as “caring,” “sympathetic,” and “attentive” are typically associated with femininity, whereas agentic attributes such as “authoritative,” “active,” and “confident” are typically associated with masculinity (12, 30).

Whereas the above two widely examined dimensions focus on generic language rather than language used specifically in hiring and labor market processes (11, 12, 18, 19), we drew on sociology, labor economics, and management research to consider four further dimensions of gender/EDI language that are more specifically salient in the labor market context:

3. *Gendered work roles* capture words describing skills and responsibilities expected of a job holder that are often constructed and perceived in a gendered way. For example, “soft” and “social” skills are typically associated with femininity vs. time-compressed and stressful roles, such as those involving “multitasking,” “pressure,” and “speed,” are typically associated with masculinity (13, 31, 32).
4. Family responsibilities play a prominent role in shaping gendered labor force participation. Thus, we capture *work–family cues* that signal support for or constraint of family responsibilities (33–39): e.g. “parental leave,” “flexible” work, and “work–family balance” (family-friendly, feminine) vs. “irregular” and “long work hours” (family-unfriendly, masculine).
5. *EDI policy* captures direct references to EDI legislation, regulation, and initiatives, such as “the Equality Act,” “Stonewall,” “Racial Equality Charter,” and “Equal Opportunity Employer” (9, 40, 41). These references speak to trends toward EDI legislation and regulation in many countries, which have increasingly encouraged employers to make EDI policy pledges in job ads (9, 41).
6. *EDI culture* captures words that describe workplace culture as egalitarian, diverse, and inclusive, such as “supportive,” “accessible,” and “empowering.” Language signaling EDI culture reflects the diffusion of EDI as an organizational ethos, going beyond mere pledges of adherence to EDI policies (1, 42).

To quantify these dimensions of language, we used the natural language processing technique of word embedding to capture not only words in our inventory but also related words with similar semantic meanings (16–19). For the first four gender dimensions, we measured the extent to which the wording of a job ad leaned toward the masculine or feminine orientation. For the latter two EDI dimensions, we measured the prevalence of EDI policy/culture language in each job ad. Within each dimension, we scaled the language scores across all ads to range between 0 (most masculine/least pro-EDI) and 100 (most feminine/most pro-EDI).

We applied the inventory to a dataset of 28,609,485 unique UK job ads posted between January 2018 and June 2023, collected by Lightcast—one of the largest organizations that monitor online job ads internationally (https://lightcast.io). Validation shows that the dataset comprehensively captures job ads posted on employer websites, major job platforms (e.g. Reed), and aggregator platforms (e.g. Monster) that collate job ads from multiple sources (43, 44). We focused our analysis on the title and main text for each job ad, as these sections play a prominent role in shaping readers’ first impression of a job, thus determining whether they seek further information about and apply for the job. See SI Appendix, Supplementary Material 4 for the methods used for calculating the language scores and attendant descriptive statistics.

### How gender/EDI language in job ads shapes labor force gender/racial composition

**Fig. 2.**
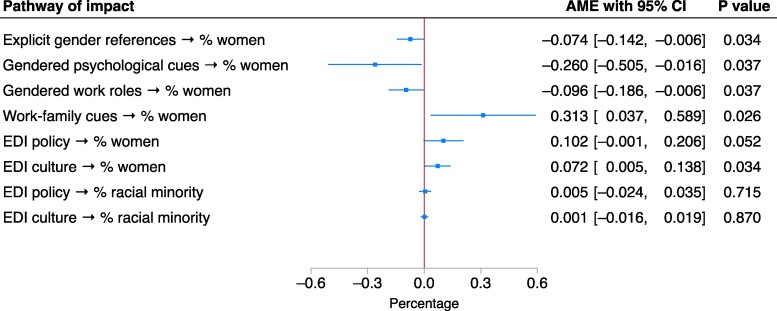
Average marginal effects of gender/EDI language in job ads on labor force gender/racial composition. See SI Appendix, Supplementary Material 7, Table S11 for model results.

In Fig. 2, we present the estimated impact of each dimension of gender/EDI language in job ads on labor force gender/racial composition. Accounting for potential bidirectional relationships between language in job ads and labor force composition, we estimated two-stage IV regression models to help mitigate endogeneity and reverse causality (27). In the models, we included the percentages of women/racial minority workers across the 189 industry-occupation groups in 2018–2023 as the dependent variable, the scores for each dimension of gender/EDI language across the 28.6 million job ads in the same period as the predictor, and the word count of each job ad and its squared term as first-stage IVs. The model also controlled for the year, region, and source (e.g. employer website, recruiter websites) of job ads. We modeled each dimension of language separately. We calculated the 95% CI based on standard errors clustered across the 189 industry-occupation groups, as the job ads were nested within these groups (45). See SI Appendix, Supplementary Material 5 for full information on the IVs and IV test results, Supplementary Material 6 for details of our modeling strategy and control variables, and Supplementary Material 7 for full model results.

Gendered language in job ads has mixed impacts on labor force gender composition. On the one hand, feminine as opposed to masculine language in job ads may deter female job seekers. In terms of explicit gender references, a 1-percentile movement from the use of explicitly masculine to feminine (pro)nouns translates into a 0.074 percentage-point decrease (95% CI: −0.142, −0.006, *P* = 0.034) in the share of women across the 189 industry-occupation groups. With a 1-percentile movement from masculine to feminine psychological cues and language associated with work roles, the share of women in the workforce decreases by 0.260 (−0.505, −0.016, *P* = 0.037) and 0.096 (−0.186, −0.006, *P* = 0.037) percentage points, respectively. On the other hand, work–family cues that signal a family-friendly orientation have a positive influence on the share of women in the workforce. With every 1-percentile movement on the scale of work–family cues from family-unfriendly (masculine) to family-friendly (feminine), the share of women in the workforce increases by 0.313 (0.037, 0.589, *P* = 0.026) percentage points.

When it comes to EDI language, the positive impact [*B* = 0.102 (−0.001, 0.206), *P* = 0.052] of EDI policy pledges on the share of women in the workforce is only statistically significant at the 10% level. Language describing workplace EDI culture has a positive impact on the share of women in the workforce. With every 1-percentile increase in the use of language that signals workplace EDI culture, the share of women in the workforce increases by 0.072 (0.005, 0.138, *P* = 0.034) percentage points. Compared with men, therefore, women appear more likely to respond positively to language signaling workplace EDI culture.

In terms of labor force racial composition, language pertaining to neither EDI policy nor EDI culture has an impact on the share of racial minority workers in the workforce, as the effects are all close to zero and not statistically significant. Despite extensive policy, regulatory, and organizational efforts at communicating EDI policies and culture in job ads (1, 9), such efforts do not seem to have any bearing on racial minority representation in the labor force.

### How labor force gender/racial composition shapes gender/EDI language in job ads

**Fig. 3.**
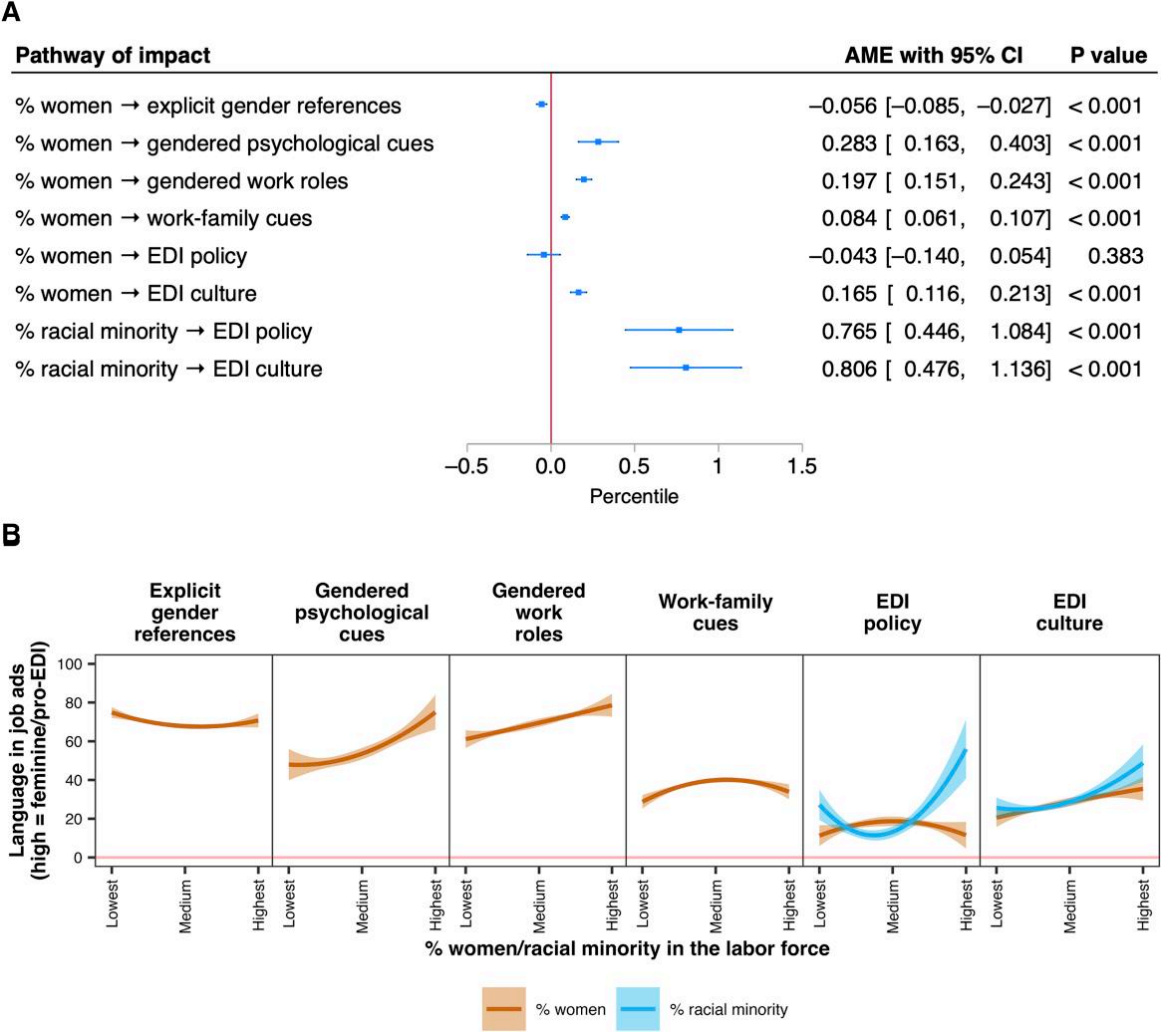
Average marginal effects of labor force gender/racial composition on gender/EDI language in job ads. Linear effects in A) and nonlinear effects in B). See SI Appendix, Supplementary Materials 7, Tables S12 and S13 for model results.

Figure 3 presents the estimated impact of labor force gender/racial composition on gender/EDI language in job ads, with 95% CI. As in the previous section, we used two-stage IV regression models to mitigate potential bidirectional relationships between labor force composition and language in job ads. In the models, we included the predicted values of each dimension of gender/EDI language for the 189 industry-occupation groups as the dependent variable, adjusting for the year, region, and source of job ads. We used the percentages of women/racial minority workers across the industry-occupation groups as the predictor. The first-stage IVs included lagged 2001–2002 labor force gender/racial/migrant composition measures across the first-level industry (SIC1) and occupation (SOC1) categories. Because all variables were measured at the industry-occupation or industry/occupation level, we estimated the models based on the reduced sample containing the 189 industry-occupation groups. See SI Appendix, Supplementary Material 5 for full information on the IVs and IV test results, Supplementary Material 6 for detailed modeling strategy, and Supplementary Material 7 for full model results.

Figure 3A first presents the linear effects of labor force gender composition on each dimension of gender/EDI language in job ads. Job ads for industry-occupation groups with a larger share of women tend to include fewer explicitly feminine rather than masculine (pro)nouns [*B* = −0.056 (−0.085, −0.027), *P* < 0.001]. In contrast, job ads for those with a larger share of women tend to include more feminine rather than masculine psychological, work-role, and work–family cues. With a 1 percentage-point increase in the share of women in the workforce, we found a 0.283 (0.163, 0.403, *P* < 0.001) and a 0.197 (0.151, 0.243, *P* < 0.001) percentile increase in the use of feminine rather than masculine psychological and work-role cues, respectively. Similarly, every 1 percentage-point increase in the share of women in the workforce is linked to a 0.084 (0.061, 0.107, *P* < 0.001) percentile increase in the use of family-friendly (feminine) rather than family-unfriendly (masculine) cues. As for EDI language, labor force gender composition has hardly any bearing on the inclusion of EDI policy pledges in job ads [*B* = −0.043 (−0.140, 0.054), *P* = 0.383]. In contrast, industry-occupation groups with a larger share of women are more likely to signal workplace EDI culture in job ads. With every 1 percentage-point increase in the share of women, we found a 0.165 (0.116, 0.213, *P* < 0.001) percentile increase in language signaling workplace EDI culture.

Figure 3A also reports the linear effects of labor force racial composition on EDI language in job ads. Racial minority representation in the workforce positively predicts the inclusion of EDI language in job ads. With every 1 percentage-point increase in the share of racial minority workers, we found a 0.765 (0.446, 1.084, *P* < 0.001) and a 0.806 (0.476, 1.136, *P* < 0.001) percentile increase in language associated with EDI policy and workplace EDI culture, respectively.

In Fig. 3B, we test the “compensation” hypotheses (Fig. 1) that employers word job ads to play up language associated with underrepresented identities (hypothesis 2, positive compensation) and suppress language associated with majority identities (hypothesis 3, negative compensation) in the workforce. Should the compensation hypotheses hold, we expect to see nonlinear impacts of labor force gender (orange lines) and racial (blue lines) composition on gender/EDI language in job ads. Building on the models reported in Fig. 3A, we further included the quadratic term of labor force gender/racial composition as a predictor of gender/EDI language in job ads across the 189 industry-occupation groups. Accordingly, we further included the quadratic, in addition to linear, terms of the lagged 2001–2002 labor force composition measures as first-stage IVs (46).

We found evidence of both positive and negative compensation in how labor force composition influences gender/EDI language in job ads. On the one hand, supporting hypothesis 2, positive compensation is observed in how labor force gender composition influences the use of explicit gender references (*B*_quadratic_ = 0.002, [0.001, 0.004], *P* = 0.014), and how labor force racial composition influences the use of language signaling EDI policy (*B*_quadratic_ = 0.126 [0.076, 0.177], *P* < 0.001). Compared with industry-occupation groups with a medium share of women, those with a small share of women tend to use more explicit feminine rather than masculine (pro)nouns. Compared with industry-occupation groups with a medium share of racial minority workers, language associated with EDI policy tends to be much more prevalent in job ads for those with a small share of racial minority workers. On the other hand, supporting hypothesis 3, negative compensation is observed in how labor force gender composition influences the use of gendered work–family cues (*B*_quadratic_ = −0.004 [−0.006, −0.002], *P* < 0.001). Compared with workforces with a medium share of women, those with a large share of women tend to use fewer family-friendly (feminine) as opposed to family-unfriendly (masculine) cues.

The evidence in this section reveals notable impacts of labor force gender/racial composition on gender/EDI language in job ads. Such impacts do not necessarily follow a linear translation of a workforce's gender/racial characteristics into corresponding orientations in the wording of job ads, as posited by identity theories (23). Rather, the evidence of both negative and positive compensation suggests that industry-occupation groups may take a reflective approach to writing job ads in a potential attempt to rectify workforce gender/racial segregation.

## Discussion

Understanding and tackling persistent labor force gender and racial segregation are crucial to facilitating equality and diversity in the labor market (1, 2, 8, 38). As a first point of contact between employers and job seekers, job ads “gatekeep” the labor force, and presently, there are extensive organizational, regulatory, legislative, and technical efforts being made to diversify and debias language in job ads (9, 11–14, 17, 19, 30, 41). Despite these efforts, however, previous research offers only a limited understanding of the relationships between language used in job ads and labor force composition, particularly the bidirectional relationships between the two. Consequently, the effectiveness of interventions in the wording of job ads in helping reduce labor force gender and racial segregation remains elusive.

**Fig. 4.**
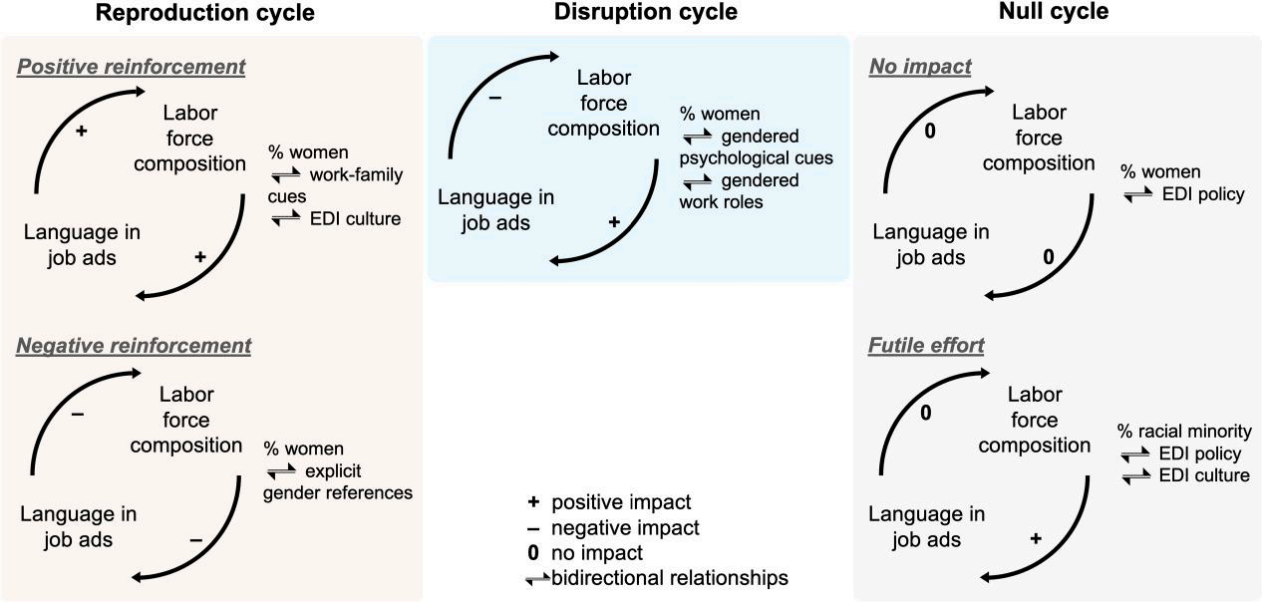
Three types of interplay between gender/EDI language in job ads and labor force gender/racial composition. Positive impact = an increase in the percentage of women/racial minority workers in the labor force leading to more feminine rather than masculine/more pro-EDI wording of job ads, or more feminine rather than masculine/more pro-EDI wording of job ads leading to a higher percentage of women/racial minority in the labor force. Negative impact = an increase in the percentage of women/racial minority workers in the labor force leading to more masculine rather than feminine/less pro-EDI wording of job ads, or more feminine rather than masculine/more pro-EDI wording of job ads leading to a lower percentage of women/racial minority in the labor force. No impact = estimated impact not statistically significant at the 5% level (based on Figs. 2 and 3).

Addressing these knowledge gaps, we systematically examined the reciprocal relationships between gender/EDI language in job ads and labor force gender/racial composition. To do so, we developed a multidimensional word inventory of gender/EDI language in job ads, crafted an IV modeling strategy to help disentangle bidirectional relationships, and leveraged natural language processing techniques in analyzing 28.6 million job ads. Our findings provide a labor-market-wide audit of (ⅰ) how gender/EDI language in job ads helps shape labor force gender/racial composition, and (ⅱ) how labor force gender/racial composition influences gender/EDI language in job ads. As synthesized in Fig. 4, taken together, our findings show that the bidirectional interplay between language in job ads and labor force composition contributes to both reproducing and disrupting gender/racial segregation in the labor market.

First, the interplay between gender/EDI language in job ads and labor force composition serves to *reproduce* labor force gender segregation through both positive and negative reinforcements. For “positive reinforcement,” job ads for workforces with a larger share of women tend to include more feminine rather than masculine work–family cues as well as language signaling workplace EDI culture. In turn, feminine work–family cues and language signaling EDI culture contribute to increasing the share of women in the workforce. For “negative reinforcement,” job ads for workforces with a larger share of men tend to include more feminine rather than masculine (pro)nouns, and such feminine (pro)nouns have a negative impact on the share of women in the labor force, thus serving to reinforce the male-dominated composition of the workforces. Our findings, therefore, uncover mechanisms through which gendered language in job ads and gendered workforce composition reinforce each other to reproduce labor force gender segregation. Moreover, our findings suggest an unintended consequence of the inclusion of EDI language in job ads (1). Insofar as female-dominated workforces are more likely than male-dominated ones to use EDI language in job ads, and insofar as female job seekers are more likely than male ones to respond positively to such language, EDI language could unintentionally serve as a vehicle of gender stratification that entrenches rather than mitigates labor force gender segregation.

Second, we also found some evidence that the interplay between language in job ads and labor force composition could help *disrupt* the reproduction of labor force gender segregation. While job ads for workforces with a larger share of women tend to include more feminine rather than masculine psychological and work role cues, such cues are found to reduce the share of women in the workforce, thus tilting the gender composition of the workforce toward a more masculine direction.

Third, our study also provides salient *null* findings regarding the absence of reciprocal relationships between some dimensions of language in job ads and labor force gender/racial composition. First, impact can be absent in both ways. For example, labor force gender composition has little bearing on the inclusion of EDI policy pledges in job ads, and such pledges have a very limited impact on labor force gender composition. Second, although workforces with a larger share of racial minority workers tend to use more EDI language in job ads, EDI language makes little difference to labor force racial composition. Furthermore, as our nonlinear results show, while workforces with a low racial minority representation also tend to adopt a positive compensation strategy and play up EDI policy pledges in their job ads, such pledges have little impact on labor force racial composition.

Despite much social, political, regulatory, and legislative emphasis on EDI and its representation in job ads (1, 9, 17, 19, 40, 41), EDI policy pledges and language signaling workplace EDI culture have no impact on workforce racial composition, for three possible reasons that should be systematically examined in future research. First, with legal and regulatory imperatives and cultural diffusion (1), EDI language and particularly policy pledges may have become so common in job ads that there is little variation across industries and occupations. Second, racial minority job seekers may view EDI claims as window-dressing institutional clichés that have limited appeal (9, 41). Third, the effects of EDI language in job ads on labor for composition may have been countervailed by intermediary procedures such as shortlisting and interviewing. The first possibility, however, seems unlikely given the relatively low prevalence of EDI policy pledges and notable variations in language signaling workplace EDI culture across industry-occupation groups (SI Appendix, Supplementary Material 4). Our findings thus call into question existing approaches to using EDI language in job ads. They urge policymakers, organizations, and HR professionals to develop meaningful and impactful ways to communicate EDI in job ads and to scrutinize the extent to which procedures such as candidate screening, shortlisting, and interviewing align with EDI claims made in job ads.

The limitations of our study suggest several directions for future research. First, we analyzed UK job ads written in the English language. Future research could expand our approach to examine job ads in other languages across a wider range of countries. Second, our findings capture the relationships between language in job ads and labor force composition at an aggregate level. This reflects our effort to go beyond previous research examining how individuals respond to gendered psychological cues under experimental conditions (11, 12, 15, 19), to provide large-scale evidence based on a labor-market-wide audit. Nonetheless, further research is needed to illuminate the process of writing and disseminating job ads (9, 13, 20). Finally, although job ads are widely used across most segments of the labor market, job search and hiring through (informal) networks, particularly for elite jobs and family businesses (47), can circumvent job ads. Nevertheless, with an increasing emphasis on fairness, transparency, and accountability, we expect informality in the hiring process to decrease, with job ads playing a prominent role in formalized hiring processes.

In conclusion, our study brings to light understudied yet important mechanisms underpinning the reproduction of labor force gender and racial segregation, by disentangling the reciprocal relationships between language in job ads and labor force composition. Although our findings highlight the bidirectional interplay between job ads and labor force composition, labor force composition cannot be changed without changing the process that selects workers into the labor force. The wording of job ads represents a crucial first step in this process. In this context, our interdisciplinary contributions—combining a novel multidimensional inventory of gender/EDI language in job ads, large-scale natural language processing, bidirectional modeling, and population-wide auditing evidence—provide a useful roadmap and toolkits for policymakers, HR practitioners, and employers to develop effective interventions. Policymakers can use our findings to frame regulatory guidelines for auditing recruitment processes, which can include assessing language used along the six dimensions we developed and examined. HR practitioners can translate such guidelines and incorporate them into professional qualification and certification criteria. As language in job ads partly reflects how the corresponding jobs are structured (e.g. irregular shifts), our findings also provide employers with clues to (re)configure jobs to be more inclusive.

## Supplementary Material

pgae526_Supplementary_Data

## Data Availability

Both the job ads obtained through Lightcast (https://lightcast.io) and the labor force data collected by the UK Office for National Statistics and obtained via the UK Data Service are copyrighted and proprietary. Access permission for the job ads data can be requested using the Lightcast online form (https://lightcast.io/contact), and the UK Labour Force Survey data can be accessed and downloaded through the UK Data Service website (https://ukdataservice.ac.uk). Full codes for data preparation and analysis are available via the Open Science Framework: https://osf.io/v8b6m. In SI Appendix, Supplementary Material 9, we provide further information on how to use our replication codes.

## Previous Presentation

An earlier version of this paper was presented at the Work-Family Researchers Network 2022 Biennial Conference in New York, USA.

## References

[1] Dobbin F, Kalev A. 2022. Getting to diversity: what works and what doesn’t. Harvard University Press.

[2] England P, Levine A, Mishel E. 2020. Progress toward gender equality in the United States has slowed or stalled. Proc Natl Acad Sci U S A. 117:6990–6997. 32229559 10.1073/pnas.1918891117 PMC7132302

[3] Ferguson J-P, Koning R. 2018. Firm turnover and the return of racial establishment segregation. Am Sociol Rev. 83:445–474.

[4] Qian Y, Fan W. 2019. Men and women at work: occupational gender composition and affective well-being in the United States. J Happiness Stud. 20:2077–2099.

[5] Quillian L, Pager D, Hexel O, Midtbøen AH. 2017. Meta-analysis of field experiments shows no change in racial discrimination in hiring over time. Proc Natl Acad Sci U S A. 114:10870–10875. 28900012 10.1073/pnas.1706255114 PMC5642692

[6] Sparber C . 2008. A theory of racial diversity, segregation, and productivity. J Dev Econ. 87:210–226.

[7] Ozgen C . 2021. The economics of diversity: innovation, productivity and the labour market. J Econ Surv. 35:1168–1216.

[8] Riccucci NM . 2021. Managing diversity in public sector workforces. Routledge.

[9] Andreassen TA . 2021. Diversity clauses in job advertisements: organisational reproduction of inequality? Scand J Manag. 37:101180.

[10] Askehave I, Zethsen KK. 2014. Gendered constructions of leadership in Danish job advertisements. Gend Work Organ. 21:531–545.

[11] Bem SL, Bem DJ. 1973. Does sex-biased job advertising “aid and abet” sex discrimination? J Appl Soc Psychol. 3:6–18.

[12] Gaucher D, Friesen J, Kay AC. 2011. Evidence that gendered wording in job advertisements exists and sustains gender inequality. J Pers Soc Psychol. 101:109–128. 21381851 10.1037/a0022530

[13] Jännäri J, Poutanen S, Kovalainen A. 2018. Gendering expert work and ideal candidacy in Finnish and Estonian job advertisements. Gend Manag Int J. 33:544–560

[14] Hu S, et al 2022. Balancing gender bias in job advertisements with text-level bias mitigation. Front Big Data. 5:805713. 35284822 10.3389/fdata.2022.805713 PMC8905631

[15] Wille L, Derous E. 2017. Getting the words right: when wording of job ads affects ethnic minorities’ application decisions. Manag Commun Q. 31:533–558.

[16] Kuhn P, Shen K, Zhang S. 2020. Gender-targeted job ads in the recruitment process: facts from a Chinese job board. J Dev Econ. 147:102531.

[17] Burn I, Button P, Corella LM, Neumark D. 2022. Does ageist language in job ads predict age discrimination in hiring? J Labor Econ. 40:613–667. 35845105 10.1086/717730 PMC9285661

[18] Chaturvedi S, Mahajan K, Siddique Z. 2021. Words matter: gender, jobs and applicant behavior. IZA Discussion Paper Series No. 14497. p. 1–67.

[19] Castilla EJ, Rho HJ. 2023. The gendering of job postings in the online recruitment process. Manag Sci. 69:6912–6939.

[20] Bills DB, Di Stasio V, Gërxhani K. 2017. The demand side of hiring: employers in the labor market. Annu Rev Sociol. 43:291–310.

[21] Neumark D . 2018. Experimental research on labor market discrimination. J Econ Lit. 56:799–866.

[22] Ozier EM, Taylor VJ, Murphy MC. 2019. The cognitive effects of experiencing and observing subtle racial discrimination. J Soc Issues. 75:1087–1115.

[23] Preece S . 2016. The routledge handbook of language and identity. Routledge.

[24] Saguy AC, Rees ME. 2021. Gender, power, and harassment: sociology in the #MeToo era. Annu Rev Sociol. 47:417–435.

[25] Lewis-Enright K, Crafford A, Crous F. 2009. Towards a workplace conducive to the career advancement of women. SA J Ind Psychol. 35:9.

[26] Bouten-Pinto C . 2016. Reflexivity in managing diversity: a pracademic perspective. Equal Divers Incl Int J. 35:136–153.

[27] Bollen KA . 2012. Instrumental variables in sociology and the social sciences. Annu Rev Sociol. 38:37–72.

[28] ‘t Mannetje A, Kromhout H. 2003. The use of occupation and industry classifications in general population studies. Int J Epidemiol. 32:419–428. 12777430 10.1093/ije/dyg080

[29] Bem SL . 1974. The measurement of psychological androgyny. J Consult Clin Psychol. 42:155. 4823550

[30] Hodel L, Formanowicz M, Sczesny S, Valdrová J, von Stockhausen L. 2017. Gender-fair language in job advertisements: a cross-linguistic and cross-cultural analysis. J Cross Cult Psychol. 48:384–401.

[31] Cook EP . 1994. Role salience and multiple roles: a gender perspective. Career Dev Q. 43:85–95.

[32] Fagenson EA . 1990. Perceived masculine and feminine attributes examined as a function of individuals’ sex and level in the organizational power hierarchy: a test of four theoretical perspectives. J Appl Psychol. 75:204–211.

[33] Kelly EL, et al 2014. Changing work and work-family conflict: evidence from the work, family, and health network. Am Sociol Rev. 79:485–516. 25349460 10.1177/0003122414531435 PMC4208075

[34] Budig MJ, England P. 2001. The wage penalty for motherhood. Am Sociol Rev. 66:204–225.

[35] Hodges MJ, Budig MJ. 2010. Who gets the daddy bonus? Organizational hegemonic masculinity and the impact of fatherhood on earnings. Gend Soc. 24:717–745.

[36] Moen P . 2003. It's about time: couples and careers. Cornell University Press.

[37] Farivar F, Cameron R, Yaghoubi M. 2016. Work-family balance and cultural dimensions: from a developing nation perspective. Pers Rev. 45:315–333.

[38] Cha Y . 2013. Overwork and the persistence of gender segregation in occupations. Gend Soc. 27:158–184.

[39] Weisshaar K . 2018. From opt out to blocked out: the challenges for labor market re-entry after family-related employment lapses. Am Sociol Rev. 83:34–60.

[40] Dickens L . 2007. The road is long: thirty years of equality legislation in Britain. Br J Ind Relat. 45:463–494.

[41] Leibbrandt A, List JA. 2018. Do equal employment opportunity statements backfire? Evidence from a natural field experiment on job-entry decisions. NBER Working Paper No. w25035. p. 1–40. https://www.nber.org/papers/w25035.

[42] Kossek EE, Lee K-H. 2020. Creating gender-inclusive organizations: lessons from research and practice. University of Toronto Press.

[43] Cammeraat E, Squicciarini M. 2021. Burning Glass Technologies’ data use in policy-relevant analysis: an occupation-level assessment. OECD Science Technology and Industry Working Papers No. 2021/05. p. 1–69. 10.1787/cd75c3e7-en.

[44] Lassébie J, Marcolin L, Vandeweyer M, Vignal B. 2021. Speaking the same language: a machine learning approach to classify skills in Burning Glass Technologies data. OECD Social, Employment and Migration Working Papers No. 263. p. 1–52. 10.1787/adb03746-en.

[45] Abadie A, Athey S, Imbens GW, Wooldridge JM. 2022. When should you adjust standard errors for clustering? Q J Econ. 138:1–35.

[46] Murray MP . 2006. Avoiding invalid instruments and coping with weak instruments. J Econ Perspect. 20:111–132.

[47] Rivera LA . 2016. Pedigree: how elite students get elite jobs. Princeton University Press.

