# Supplementary material for: Language in job advertisements and the reproduction of labor force gender and racial segregation
Source: PNAS Nexus. 2024 Nov 20;3(12):pgae526. doi: 10.1093/pnasnexus/pgae526 (PMC11630757; doi:10.1093/pnasnexus/pgae526)
Supplement: pgae526_Supplementary_Data [file pgae526_supplementary_data.pdf]

Supporting Information

Language in job advertisements and the reproduction of labor force gender and racial segregation

Yang Hu[a, *], Nicole Denier[b, *, 1], Lei Ding[c, *, 1], Monideepa Tarafdar[d,e, *], Alla Konnikov[f], Karen D. Hughes[b, g, h], Shenggang Hu[i], Bran Knowles[j], Enze Shi[c], Jabir Alshehabi Al-Ani[k], Irina Rets[e, l], Linglong Kong[c], Dengdeng Yu[m], Hongsheng Dai[n], Bei Jiang[c]

[a] Department of Sociology, Lancaster University, UK
[b] Department of Sociology, University of Alberta, Canada
[c] Department of Mathematical and Statistical Sciences, University of Alberta, Canada
[d] Isenberg School of Management, University of Massachusetts Amherst, USA
[e] Department of Management Science, Lancaster University, UK
[f] Department of Social Sciences, Concordia University of Edmonton, Canada
[g] Department of Strategy, Entrepreneurship, and Management, University of Alberta, Canada
[h] Diana International Research Institute, Babson College, USA
[i] Department of Statistics, University of Warwick, UK
[j] School of Computing and Communications, Lancaster University, UK
[k] Department of Computer Science and Data Science, York St. John University, UK
[l] Institute of Educational Technology, The Open University, UK
[m] Department of Management Science and Statistics, Alvarez College of Business, University of Texas at San Antonio, USA
[n] School of Mathematics, Statistics and Physics, Newcastle University, UK

* To whom correspondence should be addressed: Email: yang.hu@lancaster.ac.uk, nicole.denier@ualberta.ca, lding1@ualberta.ca, mtarafdar@isenberg.umass.edu

[1] Denotes equal authorship.

This file includes:

Supplementary Materials 1–9
Figures S1 to S2
Tables S1 to S18
SI References

Supplementary Materials 1: Contextual information on the labor market and job advertising in the United Kingdom

Gender and racial segregation characterize many labor markets around the world. Across all Organisation for Economic Co-operation and Development (OECD) countries, women are less likely to participate in the labor force than men, and work in distinct occupations and industries, particularly care and service sectors (1). Despite progress toward equality over the 20th century, including the dismantling of legal segregation, narrowing of gender wage gaps, and the entrance of many women into professional occupations, inequalities persist and have sparked global policy interest in areas ranging from women's career advancement to the hampering of long-term productivity growth (1). Similarly, in many countries, immigrants and racial and ethnic minorities face significant barriers to obtaining adequate employment or reaching the highest-paying or high-status occupations (2). Racial and ethnic segregation and inequality tend to reflect country-specific histories of immigration and racialization, resulting from immigration pathways explicitly tied to labor market positions, racial and ethnic discrimination, and/or network differences, among other mechanisms (3, 4). As such, governments and organizations across the world have worked to address labor market gender and racial segregation and build more equitable labor markets through national legislation and private and public sector organizational initiatives.

One such intervention focuses on attracting diverse applicants through disseminating job advertisements (ads) widely, and in ways that encourage diverse groups to apply. Across the world, job advertising increasingly takes place online on job posting, recruitment, and search platforms (5, 6). Such online job advertising may play a critical role in shaping opportunities in the labor market and ultimately the composition of the labor force by (a) disseminating information to relevant applicant pools; (b) encouraging formalized positions that bring together distinct skillsets; and (c) framing and advertising positions in language that appeals or deters certain groups of applicants, including those with and without protected characteristics. Organizations are increasingly attuned to biased language in ads and using strategies to de-bias language or include explicit statements expressing commitment to Equity, Diversity, and Inclusion (EDI) or compliance with equality legislation (7).

The United Kingdom (UK), like many other liberal economies such as the United States (8), fits squarely within this global pattern, with a labor market that is highly segregated by gender, race, and ethnicity, coupled with a sustained interest in fostering EDI. Reflecting unique histories of labor force engagement, marginalization, and inequality, women and racial/ethnic minorities face distinct labor market challenges in the UK. Women participate in the labor market at lower rates than men, are more likely to be employed part-time and work in unique occupations, industries, and sectors of employment (9, 10). Recent statistics indicate that women account for the vast majority of workers in healthcare, social services, and education sectors, but represent a small minority in primary and secondary industries like mining and construction(10). As a result of this industry segregation, women are much more likely than men to be employed in the public sector. Occupations are similarly highly segregated by gender. Men remain more likely to work in management and skilled trades occupations, while women are more likely to work in care, administrative support, and sales occupations (10). Notably,

women have made significant inroads into professional occupations and are almost as likely as men to work in professional occupations (10).

Racial and ethnic differences in the labor market also abound. In the UK, racial and ethnic differences are commonly described in terms of the official "ethnic group" classification in the Census, which are a mix of country of origin (typically associated with ethnicity) and skin color distinctions (typically associated with racial classification), including Asian or Asian British (Indian, Pakistani, Bangladeshi, Chinese or other); Black, Black British, Caribbean or African; Mixed; White (British, Irish, Gypsy or Irish Traveler, Roma, and other white groups), and other ethnic groups (e.g., Arab) (11). While some ethnic and racial minorities (compared to White British) have gained footholds in the labor market, with high rates of employment, and in high-paying occupations and industries, many ethnic and racial minorities face higher rates of unemployment, and limited access to stable and lucrative forms of employment (12, 13). For instance, while White British people are fairly evenly distributed across occupational groups, African, Bangladeshi, and Pakistani groups are highly segregated (14). Evidence from field experiments suggests that this reflects both ethnic and racial discrimination (15). Coupled with gender inequality, racial and ethnic minority women tend to have worse labor market outcomes than White British women and men, and racial and ethnic minority men occupy lower status positions than white British men (12, 13, 16).

Given these disparities, the UK government and many public and private sector organizations have implemented policies to encourage EDI in the labor market and workplaces. The Equality Act 2010 prohibits discrimination and harassment based on certain characteristics, including sex, gender reassignment, marriage and civil partnership, pregnancy and maternity, and race (17). Under the Act, the public sector has an additional duty to advance equality of opportunity between persons with and without protected characteristics. Language in job ads may offer a key avenue for advancing EDI, both through attracting diverse candidates and/or signaling commitments to the UK's national legislation.

Supplementary Materials 2: Measuring labor force gender/racial composition

2.1. Labor force data

We used data from the UK Quarterly Labor Force Survey (LFS) (https://beta.ukdataservice.ac.uk/datacatalogue/series/series?id=2000026#), which provide reliable information on the UK labor force. Specifically, we used LFS data between January 2018 and June 2023 to capture current labor force gender/racial composition and data between April 2001 and December 2002 to capture lagged labor force composition as IVs. The first quarter of the 2001 LFS was not used because it did not include comparable ethnicity measures vis-à-vis the 2018–2023 LFS. The dataset for calculating labor force composition in 2018–2023 contains 782,189 working respondents who provided valid information on their industry, occupation, gender, and ethnicity, and that for calculating labor force composition in 2001–2002 contains 430,358 working respondents who provided valid information for our focal variables. The weights provided as part of the LFS were used to adjust for sampling design and non-response bias such that our results are representative of the UK working population.

2.2. Labor force gender/racial composition measures

To calculate the labor force composition measures, we first distinguished 21 major industrial sectors at level 1 of the 2007 Standard Industrial Classification (SIC) and 9 major occupational categories at level 1 of the 2010 Standard Occupational Classification (SOC). We created 189 industry-occupation groups by cross-tabulating these major industry and occupation categories, as gender/racial composition for the same occupation category can vary considerably across different industries (18). We did not use more detailed industry and occupation classifications to ensure sufficient cell sizes for reliable analysis. Detailed lists of the industry and occupation categories are presented in Section 2.3 below.

Labor force gender composition was measured as the percentage of women as opposed to men in each of the 189 industry-occupation groups. We limited our measurement of gender to the male and female sexes as this measurement speaks to how occupational gender segregation is conceptualized and operationalized in previous research (9). However, we recognize that future research could extend our analysis to consider a more diverse range of gender identities. For labor force racial composition, we measured the percentage of racial minority as opposed to white workers in each of the 189 industry-occupation groups. The racial minority category includes all ethnic groups other than white British, Irish, and other white groups, as defined by the UK Office for National Statistics (11). This operationalization highlights visible racial traits (e.g., skin color) underlying racial segregation, discrimination, and inequality in the labor market (19). Tests showed that our results are robust to measuring racial composition using the Blau index (20) capturing ethnic diversity in each industry-occupation group across six major ethnic categories—the index captures the probability that two randomly selected individuals belong to different ethnic groups (11) (white British, white Irish, other white, mixed, Indian, Pakistani, Bangladeshi, Chinese, other Asian, Black African/Caribbean/Black British, and other; see *SI Appendix, Supplementary Materials 8, Table S14*).

2.3. Descriptive statistics for labor force composition measures

Table S1 presents the descriptive statistics for the labor force composition measures.

Table S1. Descriptive statistics for labor force gender/racial composition

**Industry-occupation group – SIC level 1, SOC level 1 (*N* = 189)**	**Mean/SD/Skewness/ Kurtosis**	**Histogram (percentage)**
% women in labor force	42.452/24.037/0.084/1.988	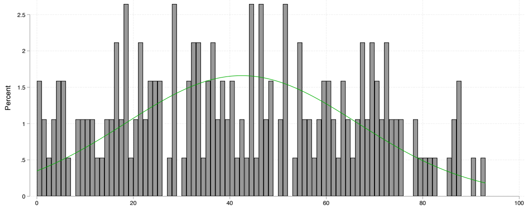
% racial minority in labor force	11.598/6.432/0.854/4.767	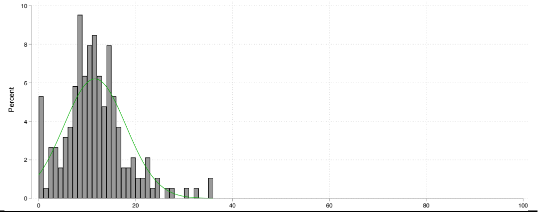

Table S2 and Fig. S1 present the labor force composition scores for each of the 189 industry-occupation groups used in the analysis. As the results show that despite long-term progress toward gender and racial equality at work, labor force gender and racial segregation persists in the UK, as in many other countries (1, 9, 15, 19). As the gender/racial composition of the same occupation (e.g., managers) varies across different industries (e.g., education vs. manufacturing), we position occupations in their industrial settings by creating 189 industry-occupation groups based on the cross-tabulation between the first levels of the Standard Industry Classification 2007 (SIC1) and Standard Occupation Classification 2010 (SOC1) (18).

Table S2. Percentages of women/racial minority workers across 189 industry-occupation groups

Industry (SIC	Occupation (SOC level1)								
level 1)	1	2	3	4	5	6	7	8	9
**% women**									
A	28.60	33.18	44.41	87.64	17.51	71.02	54.95	18.48	31.23
B	21.76	19.95	18.65	72.98	2.63	62.56	37.21	0.72	4.09
C	23.41	23.00	31.81	70.14	8.13	56.78	50.05	21.19	28.32
D	20.12	17.63	33.89	69.51	3.51	42.77	51.30	10.92	9.60
E	25.79	28.92	31.91	73.28	4.35	23.60	51.54	5.62	5.94
F	16.99	16.21	33.42	80.83	1.17	38.04	44.77	1.90	4.50
G	32.57	40.95	45.48	71.25	15.15	67.22	63.42	15.52	36.13
H	21.70	28.89	25.27	58.59	3.75	55.43	54.33	5.73	24.39
I	46.30	50.08	58.36	72.34	30.51	74.38	59.47	18.09	61.54
J	27.27	22.52	36.15	70.17	9.42	64.42	46.31	18.19	42.13
K	33.46	32.93	36.67	69.91	6.91	70.31	60.43	21.67	44.03
L	46.17	38.10	59.21	78.96	8.97	47.87	67.15	13.49	40.76
M	37.41	35.62	48.30	73.32	11.39	79.85	56.77	16.28	34.18
N	39.03	38.57	46.50	72.57	14.92	63.73	48.60	11.48	51.66
O	44.08	53.46	41.65	69.53	14.34	75.72	63.74	16.55	47.37
P	54.20	66.55	64.58	87.10	51.81	86.36	66.55	25.88	87.09
Q	68.40	74.86	72.53	86.97	43.90	82.55	67.06	20.07	67.94

R	36.88	48.77	44.24	69.23	18.21	51.74	60.63	10.33	54.62
S	55.52	40.17	59.08	75.69	12.61	81.95	65.51	24.75	57.99
T	61.28	68.78	78.99	85.38	28.13	90.84	32.44	23.70	92.18
U	32.46	35.09	24.28	60.51	0.00	39.47	46.85	0.00	35.30
**% racial minority**									
A	0.98	2.77	3.34	0.00	0.59	1.12	7.75	2.48	2.44
B	3.50	15.23	5.10	4.23	7.55	0.00	13.92	0.72	0.00
C	6.85	11.82	7.75	8.10	5.78	15.11	10.54	10.71	12.85
D	8.40	12.15	9.43	9.82	7.54	0.00	13.97	15.91	17.52
E	5.14	6.67	8.19	7.44	2.64	6.82	9.88	6.33	8.66
F	6.89	11.60	7.29	8.16	4.77	7.65	8.76	6.04	7.05
G	12.52	24.01	10.12	9.39	8.06	16.07	14.43	9.51	14.17
H	11.20	14.41	10.85	15.26	8.10	14.76	22.67	24.94	18.08
I	15.87	13.61	13.74	13.42	23.23	10.65	22.58	30.49	15.08
J	14.19	21.51	14.33	15.53	13.86	16.50	11.76	12.48	13.10
K	16.24	22.51	13.57	12.39	9.87	8.04	14.81	10.10	20.00
L	11.39	15.22	11.50	14.75	5.06	14.39	10.72	8.96	8.10
M	8.55	14.51	12.27	14.30	10.79	4.58	16.06	10.05	14.27
N	10.68	16.43	11.10	13.30	3.19	17.80	12.85	11.18	20.28
O	8.73	14.72	8.80	11.92	10.47	19.02	12.87	9.64	12.83
P	9.52	11.41	10.54	8.95	9.78	11.05	17.68	11.95	15.22
Q	11.23	22.86	14.10	11.54	10.51	19.23	11.07	7.82	18.02
R	7.51	8.44	8.95	5.67	3.71	6.82	14.90	5.63	8.29
S	12.17	12.24	12.08	10.98	10.03	9.13	18.05	7.76	19.52
T	0.00	35.14	16.01	9.56	3.15	27.63	0.00	9.35	11.68
U	19.14	21.69	15.33	32.93	16.03	0.00	26.90	35.14	2.68

Note: Calculated based on pooled Quarterly Labor Force Survey data between January 2018 and June 2023. SIC = Standard industry classification 2007, where A = Agriculture, forestry and fishing, B = Mining and quarrying, C = Manufacturing, D = Electricity, gas, steam and air conditioning supply, E = Water supply, sewerage, waste management and remediation activities, F = Construction, G = Wholesale and retail trade; repair of motor vehicles and motorcycles, H = Transportation and storage, I = Accommodation and food service activities, J = Information and communication, K = Financial and insurance activities, L = Real estate activities, M = Professional, scientific and technical activities, N = Administrative and support service activities, O = Public administration and defence; compulsory social security, P = Education, Q = Human health and social work activities, R = Arts, entertainment and recreation, S = Other service activities, T = Activities of households as employers; undifferentiated goods- and services-producing activities of households for own use, U = Activities of extraterritorial/international organizations and bodies. SOC = Standard occupation classification 2020, where 1= Managers, directors, and senior officials, 2 = Professional occupations, 3 = Associate professional and technical occupations, 4 = Administrative and secretarial occupations, 5 = Skilled trades occupations, 6 = Caring, leisure and other service occupations, 7 = Sales and customer service occupations, 8 = Process, plant, and machine operatives, 9 = Elementary occupations. Racial minority refers to all ethnic groups other than white British, white Irish, and other white groups.

In Fig. S1, the left panel depicts the percentage of women in each industry-occupation group, with a lighter color indicating a larger share of women as opposed to men. There is clear evidence of occupational gender segregation. While managerial, professional, and manual (e.g., skilled trade) occupations are largely dominated by men, service occupations (e.g., care, leisure, sales) are female-dominated. There is also notable industrial gender segregation:

whereas manual (e.g., mining and quarrying, construction) and STEM (science, technology, engineering, and mathematics) industries tend to be male-dominated, industries such as education, health, and social work are female-dominated. Considering industry-occupation intersections further reveals additive gender segregation. For example, 72.7% of managers in the information and communication industry and 98.8% of skilled-trade workers in construction are men, whereas 87.1% of administrators in education and 82.6% of care and service workers in health and social work are women.

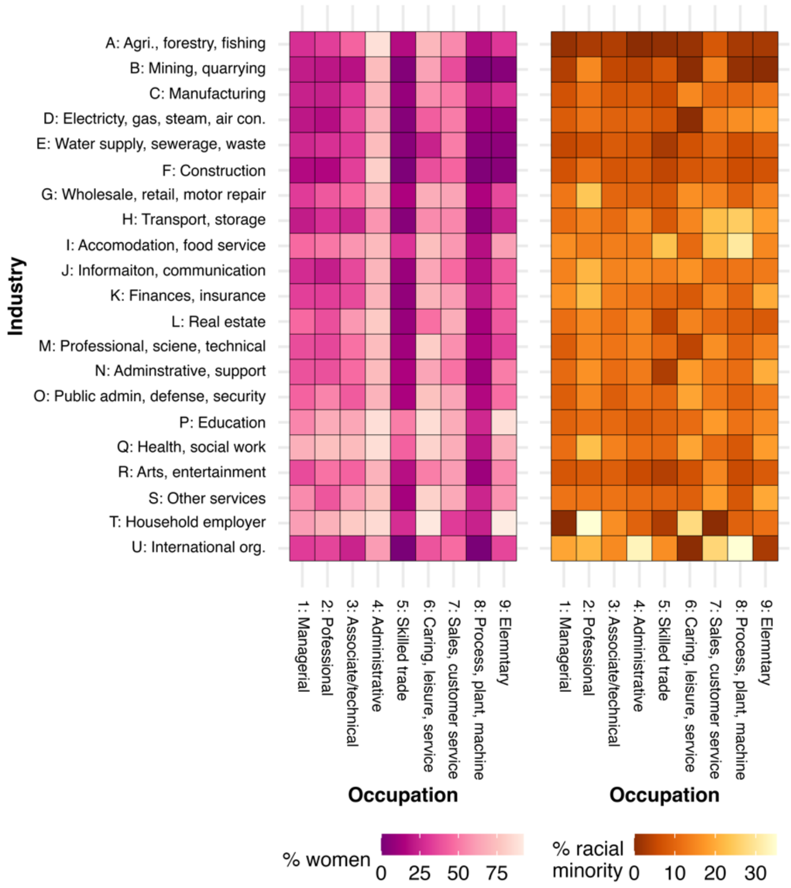

Fig. S1. Gender and racial compositions of industry-occupation groups. Calculated based on 782,189 working respondents across 189 industry-occupation groups from the Office for National Statistics Quarterly Labor Force Survey between January 2018 and June 2023. Industry is measured at level 1 of the Standard Industry Classification (SIC), and occupation is measured at level 1 of the Standard Occupation Classification (SOC). White racial identity includes white British, white Irish, and other white ethnic groups, and racial minority identity refers to all other ethnic groups.

The right panel of Fig. S1 depicts the percentage of racial minority workers in each industry-occupation group, with a lighter color indicating a higher share of racial minority as opposed to white (white British, white Irish, and other white) workers. Small occupational differences are noted, with the percentage of racial minority workers ranging from 3.8% (skilled trade) to 7.6% (sales and customer service). Greater variations are found across different industries, with the percentage of racial minority workers ranging from 0.9% (agriculture,

forestry, fishing) to 16.9% (international organizations). Some industry-occupation groups have a particularly high concentration of racial minority workers, including administrators in international organizations (32.9% racial minority) and process, plant, and machine occupations in accommodation and food service sectors (30.5% racial minority). By contrast, there are hardly any racial minority managers in agricultural, forestry, and fishing industries or racial minority elementary workers in mining and quarrying.

The patterns reported in Table S2 and Fig. S1 are not entirely surprising, as they echo historical records of labor force gender and racial segregation. Nonetheless, our findings highlight the continuing relevance of examining gender and racial segregation in the UK labor force.

Supplementary Materials 3: A multidimensional word inventory of gender/EDI language in job ads

Table S3 presents an illustration of our multidimensional word inventory of gender/EDI language. In this section, we elaborate on the background, theoretical and empirical underpinnings and the methodological procedures for developing and validating the inventory.

Table S3. A multidimensional inventory of gender and EDI language in job ads—An illustration

Dimensions	Example lexicons	Example job advertisement excerpts
Explicit gender references	*Masculine*: he, men, his<br>*Feminine*: she, he, women	● Jenna discovered collaboration working at [company name]. Playing off everyone's unique strengths and utilizing them to reach shared goals is what Jenna loves most about **her** work at [company name].<br>● The use of the **masculine** in our communications refers equally to both **men** and **women**.<br>● The supervisor is responsible to manage, organize, prioritize and direct the operations of **his** or **her** department to meet production schedules.
Gendered psychological cues	*Masculine*: confident, effective, innovative, (pro)active, practical, pragmatic, problem-solving	● **Confident** to work with **high-calibre** people.<br>● Proven ability to be **effective** in a fast-paced, ambiguous, and changing environment.<br>● Encourage new ideas and **innovative** approaches and **actively** share knowledge and experience to enhance the development of the team.
	Feminine: attentive, accurate, timely, caring, polite, diplomatic	● You have strong **attention to detail**.<br>● Responsible for the **timely** and **accurate** maintenance of accounting systems at [town name].<br>● High level of initiative, maturity, **tact** and **diplomacy**.
Gendered work roles	*Masculine*: multi-task, pressure, speed, stamina, stress	● Manage multiple competing projects and priorities under **time pressure**.<br>● **Multi-tasks**, sets appropriate priorities and acts every day with a sense of urgency and **speed** to move the organization forward.<br>● As a [company name] worker you will be faced with **stressful** situations.
	Feminine: social skills, collaborate, communication	● Strong written and oral **communication** skills.<br>● Ability to maintain **organised**, well-documented records.<br>● Proficient **interpersonal** and client services skills, ability to effectively **communicate** and **collaborate** with all levels of the organization.
Work-family cues	*Masculine (family-unfriendly)*: travel, after hours, nights, weekends	● This role is based in [place name] with **travel** throughout North America and some global **travel** required.<br>● Hours will vary and will include **evening** and **weekend** work.<br>● You are prepared to work **nights** and **weekends**, as required.
	Feminine (family friendly): part-time, paternity (leave), flexible, regular hours, work-life balance	● We promote **work-life balance**.<br>● Benefits: […] **maternity** and **parental leave benefits,** vacation (4 weeks), personal and **family leave** days.<br>● [company name] offers employees … **flexible family-friendly** programs and opportunities for professional and personal development.
EDI policy	Equality Act, specific minority identities	● No terminology in this advert is intended to discriminate against any of the protected characteristics that fall under the **Equality Act 2010**.

		● [Company name] is an **Equal Opportunity** employer.<br>● **Equality of opportunity is our policy**.
EDI culture	Work environment, advance careers, benefits	● We offer … a **pleasant** work environment and benefits package.<br>● We are committed to providing an **inclusive** and **barrier-free** work environment, starting with the hiring process.<br>● We have a **supportive**, family-oriented group of staff that are great to work with.

Note: EDI = Equality, diversity, and inclusion. Focal words/phrases are highlighted using bold font.

3.1. Background

It is clear from existing research that language used in job ads plays a central role in shaping how job seekers respond to the advertisements. Now classic studies, such as Bem and Bem (21), show how gendered language in job ads discourages applicants of the opposite sex from applying for jobs. Subsequent studies have also shown how implicit gender preferences are signaled through the use of specific traits and behavioral terms (22). Against this backdrop, we developed a systematic multidimensional word inventory for capturing gender and EDI cues in labor market texts including job ads.

To contextualize our contribution, we begin Section 3.2 with a brief review of existing word inventories, noting their strengths but also the limitations of social and cognitive psychological perspectives that inform much work in this area. We also outline our own multidisciplinary approach, building on sociology, labor economics, management, and organization studies, that is attuned to context and the social construction of gender and ethnicity/race as they relate to inequalities in the labor market. In Section 3.3, we discuss our methodological approach, which relies on expert coding of actual job ads, and iterative cycles of inter-rater comparison and validation. Following a "toolkit" approach in Section 3.4, we introduce our word inventory, which offers comprehensive coverage of gender/EDI words/phrases that may reflect employers' preferences with respect to characteristics including the gender and racial/ethnic identities of potential applicants. In addition to supporting the analyses represented in this Article, our word inventory promises broader relevance for researchers working on analyses of labor-market-related languages. In Section 3.5, we discuss the limitations and broader use of our word inventory beyond the context of this research.

3.2. Existing inventories and our approach

There are two dominant approaches to developing inventories of biased language. The first is rooted in psychological understandings of sex/gender roles and personality traits and identifies words traditionally associated with masculine or feminine characteristics (i.e., caregiving, assertiveness; agentic vs. communal) (23, 24). The Bem Sex-Role Inventory, for instance, is a list of words associated with masculinity and femininity identified through studies where participants indicated words or traits that are desirable for men or women (25, 26). Further research has demonstrated the importance of such word choices for attracting or deterring applicants to certain jobs, and that gender-biased language varies according to the level of male dominance in an occupation or level of gender inequality in a country (25, 27, 28). The second approach relies on examining the linkages between gendered names, pronouns and nouns, and language, often using a large corpus to identify word associations (29). Recently, scholars have begun to integrate these methods. For instance, Cryan and colleagues combine lexicon-based

and word association approaches, first identifying target words, then using crowdsourced participants to score thousands of words for masculinity and femininity, and finally using supervised learning to identify additional biased words in a larger corpus (23).

While these approaches are useful in conceptualizing and identifying biased language, they are necessarily limited. First, both approaches thus far have been used to focus on a singular dimension of inequality, most often gender. Second, both offer a “one-size-fits-all” list of words, failing to account for differences in language used in different social settings. For instance, the contents of a word inventory may not be exhaustive of the ways employers subtly signal gender preferences or preferences related to other employee identities such as ethnicity and race. Yet, corpus-based approaches highlight biased text across a generic range of contexts (e.g., the Wikipedia) such that identified biased words might not be locally meaningful, especially in formalized and regulated labor markets. Together, both fall short of explicitly attending to contextual cues in language. These gaps motivate our proposed method.

The method we outline below builds on and goes beyond psychological approaches to identify contextually relevant social cues in job ads that may signal preferences for certain types of workers. We rely on expert coding conceptually informed by research in sociology, labor economics, and management and organization studies. By relying on expert qualitative coding rather than mere empirical associations of words and generic gender roles, we are also able to identify concepts and words linked to other forms of categorical inequality, such as those based on race and ethnicity. There are well-established separate bodies of research on the labor market outcomes of women and racial minorities that point to unique barriers and some shared challenges in the labor market. Our approach is attuned to how job ads may convey these unique and shared challenges, as discussed in further detail below.

3.3. Method for creating and validating the word inventory

Our analysis is informed by qualitative coding methodology—the process of labeling and mapping the meanings and messages in text (30). The deductive nature of qualitative coding facilitates an in-depth and embedded understanding of the messages conveyed in the text (31). These meanings are grounded within the specific context in which these texts are produced and read (32). Our analysis of the cues that appear in job ads is rooted in the expertise of social scientists in our team and scholarship on labor market inequality connected to gender, ethnicity, race, and broader EDI issues. A similar approach of relying on academic literature for generating word embeddings was reported by Manzini and colleagues (33).

Informed by the conceptual framework outlined above, our methodology for producing a word inventory for identifying gender/EDI words/phrases in job ads comprised several steps, including preliminary qualitative coding, conceptual analysis of the corpus of preliminary codes, and inter-coder verification and finalization. First, we derived a randomly-selected sample of 160 real job ads from our dataset of job ads. Using the sample, the team, building on their disciplinary expertise in employment and organization, immigration, racial/ethnic relations, gender, work and family, conducted a preliminary qualitative content analysis to identify cues associated with gender, ethnicity, citizenship, work-family balance, and EDI. Next, the team performed a conceptual analysis of the preliminary coding, grounded in the labor market inequality literature. The final phase included an inter-rater reliability verification and close collaboration with computer scientists to refine the corpus and adapt it to algorithmic use. At this

stage, words (or word roots) that have extremely low frequencies of appearance (less than 20 counts) in our large database of 28.6 million job ads were eliminated from the inventory. The next sections elaborate on each of the steps in detail.

3.3.1. *Preliminary qualitative coding*

From our main corpus of job ads, a random sample of 160 real-data job ads was generated using the Mersenne Twister random function in Python. Four social scientists in the research team each manually coded 40 of the selected job ads to identify all potential cues related to gender, ethnicity/race, and EDI. This preliminary coding generated a large corpus of key words/phrases that clustered across three main areas: (a) explicit gender references (such as pronouns [she/he/her/him]); (b) implicit psychological, behavioral, and cognitive cues that describe candidates and roles, such as agentic and communal traits; and (c) explicit and implicit cues that describe the workplace characteristics, work-family policies, and EDI policies.

3.3.2. *Conceptual analysis of the preliminary corpus*

The next stage involved organizing the large pool of key words/phrases generated from the preliminary coding across distinct conceptual dimensions and categories. During this phase, we conducted a multi-stage content analysis. First, we cleaned and de-duplicated the cues identified by different coders. Second, we conducted another round of coding to identify the orientations of the keywords within its conceptual cluster, such as “masculine” vs. “feminine” for gender cues and cues that “promote” vs. “hinder” work-family balance and EDI. This stage was informed by theories and existing scholarship on gender inequality, work-family relations, racial/ethnic relations, and EDI in the labor market. While during the process of identifying gendered psychological cues, we partly relied on previously documented agentic and communal psychological traits (25, 26), our analysis of cues in other dimensions such as race/ethnicity, work roles, work-family policies, workplace characteristics, and EDI policies was new.

To perform the conceptual analysis of dimensions other than explicit gender references and cognitive/psychological gender cues, we turned attention to traits typically associated with status-based forms of (de)valuation as well as explicit barriers, policies, programs, workplace environments that may hinder or encourage applicants with, for instance, different gender and racial identities. As an example, we incorporated words describing skills, such as “communicational” and “soft” skills following evidence that the notion of soft skills can lead to the exclusion of ethnic/racial minorities in the labor market (34, 35). In another example, we identified words that replicate documented structural barriers that exclude women, particularly given the gendered construction of family and care responsibilities, from male-dominated fields, such as “long hours” and “frequent trips” (36, 37). In this stage, to ensure the reliability and validity of this analysis, we combined both separate and shared iterations of analyses between the four coders, followed by discussions until a consensus on the final conceptual dimensions was reached.

3.3.3. *Inter-rater reliability, inventory cleaning, and finalization*

To ensure the scientific rigor of our word inventory, we paid particular attention to inter-rater validity and reliability, as well as computational validation. Specifically, each phase included an individual coding of data conducted by four independent coders specializing in labor market

inequalities associated with gender, work and family, and EDI in the labor market. In each round of coding, individual coding was followed by group sharing and discussion of the preliminary outcomes among the four coders. This combination of individual and group analysis allowed the team to trace the development of the word inventory, to ensure inter-rater reliability in each phase and contextualize each phase within relevant scholarly literature, policies, and definitions. The conceptual dimensions and word inventory were further validated by three additional expert coders from the team. Through a double-blind coding approach, the three additional coders independently assessed the dimensions and coding produced by the first four coders, which demonstrated a high level of consistency. The dimensions and word lists were then finalized through further deliberation across the four coders and three additional validators. Because we used an iterative multi-round coding process that represents a knowledge construction process, the inter-coder consistency rate in the developmental coding varied between 0.6–0.8. Notably, once we reached the final set of words for our inventory, the final validation by three fresh validators within the team achieved a high level of inter-coder consistency exceeding 0.8.

As a key purpose of the development of this word inventory was to ensure accurate computational analysis of gender/EDI language in job ads, the word inventory was finalized with further input from computational experts. This step included reworking the words into their "root" version to allow the identification of multiple related words with the same root and semantic meaning.

3.4. The detailed word inventory

In this section, we describe the word inventory used for calculating the gender/EDI language scores reported in the Article. The inventory is formed, at the first level, of two broad sets of words—i.e., gender language and EDI language. While inventories for the identification of gender biases have had a long tradition (for example, dating back to Bem's inventory developed in 1974), we particularly separate out the second set of EDI cues that have been less systematically identified and synthesized in existing research.

On the one hand, within the set of gender cues, we further distinguished four lists of words in terms of explicit gender references, gendered psychological cues, gendered work roles, and work-family cues, with the first two dimensions expanding on existing inventories and the latter two dimensions representing our new development. Detailed information on and theoretical underpinnings for each word list are presented below. Here, it is important to note that we removed from the list of gendered psychological cues the words that overlap with those in the Gaucher, Freisen, and Kay (25) and Bem (26) inventories. Thus, for identifying psychological cues, the inventory needs to be used in combination with the Gaucher, Freisen and Kay (doi:10.1037/a0022530) (2011) and Bem (doi:10.1037/ h0036215) (1974) inventories. On the other hand, the EDI cues cover two lists of words in terms of EDI policy (and legislation) cues, and workplace EDI culture and practice. Together, the word lists form a comprehensive word inventory for gender/EDI language in a labor market context, but each specific word list can be used on its own to probe different dimensions of labor market, human resources, and organization processes. To optimize our word inventory for computation analyses, we use the symbol "*" to indicate the cut-off for word root, and multiple database formats are provided.

3.4.1. *Explicit gender references*

Job ads may include direct references to gender, through explicit mentions of gendered nouns, pronouns, and identity markers.

Feminine	Masculine
***Words and word roots*:** gal; *women; *woman; lady; her; she; feminine	***Words and word roots*:** guy; *men, *man; his; he; masculine

3.4.2. *Gendered Psychological cues (excluding the Gaucher et al. [2011] and Bem [1974] inventories)*

The second list builds on existing, widely used psychological inventories of traits associated with gender roles. Gender inequality in labor markets is long-standing and despite changing gender relations, legislation, and economic dynamics, progress toward gender equality has largely been characterized as "stalled" (38, 39). Although women's labor force participation has increased dramatically in the last thirty years, women have made only limited and selective inroads into traditionally male-dominated occupations and face wage penalties even in highly paid occupations, contributing to persistent gender segregation and gender wage gaps {Citation}. Status-based expectations attached to gender form the standards against which people are selected, evaluated, and rewarded, which may ultimately contribute to both segregation and gender wage disparities (40, 41). For instance, women are often perceived as less committed to professional careers (42) and less suitable to perform tasks in fields that have been traditionally male-dominated (43). These expectations and organizational priorities shape employer, colleague, and customer perceptions of an "ideal worker" (44, 45). The list of words below captures psychological cues that tap into these gendered expectations.

Feminine	Masculine
Words and word roots*:** accura*; attent*; caring; collabor*; committed; communicat*; courteous; creative; dedicated; detail*; diploma*; follow*; friendly; organized; patient; persua*; polite; thoughtful; tact*; timely; welcome<br>Exact phrases:*** person*-centered; attention to detail	***Words and word roots*:** accountab*; alone; authoritative; best; busy; calm*; composed; confiden*; driven; dynamic; eager; effective*; efficient*; eloquent; empower; energ*; engag*; enthusias*; exceed; excel*; exceptional; exciting; firm; frank; fun; initiative; innovative; inspirational; limitless; motivat*; outgoing; outstanding; passion*; perseverance; persistent; practical; pragmatic; proactive; productiv*; reliab*; resilien*; resolve; resourcefulness; respected; serious; strong; talented; tenacious*; vibrant; win*<br>***Exact phrases:*** can-do; forward thinking; hard-working; high quality; high calibre; problem solv*; self-driven; self-motivated; self-starter; think outside the box; time* management

3.4.3. *Gendered work roles*

The third list focuses on the roles employers expect job applicants to carry out at work. The work practices expected of a job candidate can often be gendered. The importance of role

enactment in (re)producing gender (biases) is firmly anchored in the conceptualization that gender, as an achieved social status, is enacted through the ongoing "doing" of gender (46). In the literature on gender in the labor market, it has long been established that different work tasks and occupational contexts are often sex-typed to denote gendered traits and preferences (47, 48). The following list of words capture potentially gendered ways in which employers describe the expected roles of a worker.

Feminine
***Words and word roots*:** administrat*; communicat*; interpersonal; listening; organiz*; repetitive
Exact phrases: people skills; social skills; soft skills

Masculine
***Words and word roots*:** physical*; bend*; challeng*; cold; crouch*; demand*; driving; exert*; fit*; heavy; humid; lift*; kneel*; mov*; negotiat*; numera*; pressure; pulling; pushing; reaching; risk*; safety; precautions; speed; stamina; stoop*; stress*; transport; twisting; weight; wet; outdoors
Exact phrases: hands-on; multi-task

3.4.4. *Work–family cues*

Gender biases at work are constructed in relation to other life domains such as the family. Indeed, gender biases in job ads are often associated with the differential roles women and men assume in different-sex families. Decades of work-family scholarship clearly shows that employers' family(-friendly) policies have a significant impact on the gendered labor force participation of job candidates and employees (45, 49). Gender biases and inequalities in phenomena such as the female marriage penalty, fatherhood premium, and motherhood penalty in the labor market all reflect the importance of the work-family interface in shaping labor market dynamics and outcomes (50–52). Further, as expected roles in the family differ for individuals of different gender identities, experiences of work-family conflict and balance also vary across different genders (53). As a result, employers' flexible work, work-family balance, and family support policies, and the temporal regimes of employment schedules play significant roles in shaping work participation and experiences in highly gendered ways (54, 55). The following list includes potential cues that may invoke gendered work-family orientations and considerations, with "feminine" cues indicating those that support work-family integration (WFI) and "masculine" cues indicating those that are barriers to WFI.

Supports WFI (feminine, family friendly)
***Words and word roots*:** childcare; flextime; holiday; maternity; maternal; mother; motherhood; father; fatherhood; paternity; paternal; parenthood; parental; permanent; pregnancy; part-time; sabbatical; scheme; subsidize*; telecommute; telework; vacation

WFI challenge (masculine, family unfriendly)
***Words and word roots*:** callout; evening*; furlough; indefinite; layoff; multisite; overtime; relocation; standby; temporary; travel; urgen*; weekend*; fulltime

Exact phrases: 5 days per week; 8 am–6 pm/9 am–5 pm; childcare vouchers; comprehensive benefits; contracted; digital work; family friendly; family values; flexible benefits; flex* (+ hours, days, location, schedul*, shift*; workplace practices); guaranteed hours; job share; leave of absence; Monday – Friday; onsite daycare; onsite nursery; maternal/parental leave; paid leave; part-time; personal li*; regular hours; reinstatement rights; remote work; standard hours; time off; sick* pay; sick* leave; sick* time; spousal hire; standard hours; supportive supervisor; work at home; work from home; work-family balance; work-life balance; work/life balance; (assigned; scheduled +) shift; schedul* flexibility

Exact phrases: commission package; business travel; international travel; additional hours; after hours; call out; different areas; different locations; extra (+ hours, shifts, days); fixed term; holiday cover; live-in; location change; long hours; on call; overnight travel; on-site visits; rotating (+ days, schedul*); shift (+ work; schedul*); sickness cover; work travel; work on commission; willing* to travel; willing* to relocate; (night; weekend; holiday; evening; overnight; graveyard; closing; split; varying; rotating; rotational +) shift; atypical schedul*

3.4.5. *EDI policy*

As labor market inequality and discriminatory practices come under growing scrutiny, normative pressure, broader social movements, legislation and regulatory oversight, employers have sought to boost the presence and inclusion of “historically underrepresented” groups (56, 57). This can be seen in a range of EDI policy pledges and legal references that seek to attract specific groups of workers and/or emphasize the value of cultural diversity (58, 59).

***Words and word roots*:** accessibl*; B(A)ME; disab*; discriminat*; divers*; equal*; equit*; fair; harassment; human rights; impartiality; inclusi*; language*; LGBT+; LGBTQ+; Merit*; nationalities; neutrality; Stonewall
Exact phrases: all genders; all-qualified; barrier free; Disabilities Act (2005); Equality Act (2010); equal opportunity (employer); encourages members; free from* (discrimination/harassment); minorit* candidates; Racial Equality Charter; required by law; under-represent*; Work Act 1974

3.4.6. *Workplace EDI culture*

Beyond using language aimed at attracting specific groups of workers and emphasizing the value placed on EDI, employers also highlight the positive aspects of workplace culture and practices generally and in relation to EDI (60, 61).

***Words and word roots*:** accessib*; best; busy; care; challenging; committed; community; culture; dynamic; empower; friendly; fun; global; growth; inclusive; innovative; international; leading; limitless; mission; multi-site; multidisciplinary; pleasant; progressive; rewarding; sociable; support; supportive; team; tenure; training; vibrant; young
Exact phrases: advance careers; award-winning [team]; barrier-free; best people; best places; career advancement; career development; career progression; core values; employee

assistance; fast-paced; good relations; ideal location; internationally recognised; personal development; professional development; progression opportunities; training courses

3.5. Limitations and broader use of the word inventory

Previous research exploring the textual representation of EDI issues in the labor market, with a predominant focus on gender cues, has primarily drawn on the disciplines of social linguistics and cognitive psychology. Expanding on this tradition, our word inventory speaks directly to the broader social construction of gender, ethnicity/race, and EDI in the labor market. As a whole, our inventory provides a comprehensive coverage of gender/EDI words that may signal employers' version of an ideal worker.

The limitations of our word inventory suggest several important directions for future research and development. First, all seven researchers involved in the process of manual coding and validation were experienced in social research on EDI issues in the labor market and may thus be more sensitive to EDI language than lay readers. This means that the development of the word inventory is explicitly informed by theories and extensive empirical research on gender and EDI in the labor market.

Second, as the construction of labor market EDI policies and that of EDI culture are context-specific, it is important to note that the inventory is developed from a large corpus of English job ads from the UK. Our inventory is partly shaped by the labor market legislation and broader social and cultural configurations (e.g., pertaining to gender and ethnicity/race). The underpinning corpus is composed of job ads – an important site where textual cues relating to gender, ethnicity/race, and other EDI issues are found, which was tailored to suit our research focus on job ads. For broader use, the extent to which the inventory applies to other corpora, such as organizational regulations and documents in human resources management (e.g., those pertaining to performance evaluation, promotion, retention, etc.) will require further verification.

Its limitations notwithstanding, our word inventory can be used in, but is not limited to, the following ways:

- For computer scientists, our word inventory can be used to inform the development of word embeddings and machine learning algorithms, for the development of bias detection and mitigation tools. Traditionally, the detection and mitigation of gender, ethnic/racial, and migrant biases using machine learning and artificial intelligence tended to rely on general-purpose corpora such as the Wikipedia and newspaper articles (62, 63), which are not well suited to capture EDI language in labor market processes in particular and everyday social and organizational life in general. Built on a corpus of job ads, our word inventory is particularly suited for identifying language that is directly related to EDI issues.

- For social scientists researching labor market EDI issues, the inventory (in its totality or its sub-lists) can be used to quantify distinct dimensions of gender/EDI language in labor market processes (64). This can be achieved through a direct word search based on our inventory or through more advanced techniques such as wording embedding and machine learning (65).

- Our inventory also provides unique opportunities for interdisciplinary research on EDI-related topics. As our different sub-lists are respectively informed by the disciplines of (social) psychology, sociology, social policy, labor economics, and management and organization studies. Comparative analysis and application of the sub-lists to a given labor market process promises to reveal how the different disciplines can bring to light different aspects of EDI-related dynamics in the labor market.

Supplementary Materials 4: Measuring gender/EDI language in job ads

4.1. Methods of measuring gender/EDI language

To quantify gender/EDI language, we build on our unique multidimensional word inventory. Using the inventory, we first applied pre-processing to the 28.6 million job ads, including removing HTML tags, special tokens, and punctuations (66). While it is not viable to develop a word inventory that exhausts all gender/EDI language used in job ads, we leveraged the technique of word embedding that allows us to quantify not only the words (and phrases) directly included in our inventory, but also related words with similar gender/EDI semantic meanings that are not included in our inventory (65, 67). Compared with counting the appearances of words in a pre-defined inventory (21, 25), this approach more comprehensively captures gender/EDI language (28, 68).

Word embedding uses vector representation to capture the semantic meaning of each word (23, 65). Converting words into numeric vectors facilitates quantifying the relatedness between two vectors and the words they represent. For example, for two vectors, *w* (for a word in our inventory) and *v* (for a word not in our inventory), we can use their cosine similarity score *d*, a widely-used metric that measures the closeness between two vectors, to capture the level of similarity in the semantic directions of the two words (69, 70). A score of 1 indicates that the two vectors point in exactly the same direction, and a score of –1 indicates that the two vectors point in completely opposite directions:

$$d = \frac{w \cdot v}{||w||_2 \cdot ||v||_2}. \tag{1}$$

Here, the similarity between *w* and *v* is quantified using their inner product $w \cdot v$, which is subsequently normalized by the product of their Euclidean norms $||w||_2$ and $||v||_2$.

We developed an algorithm to calculate a score for each dimension of gender/EDI language for each job ad (23, 71). Taking gendered psychological cues as an example: the input into the algorithm includes (1) a collection of words, W, in a job ad and (2) two lists of words in our inventory, A_1 and A_2, that are associated with a masculine and a feminine orientation, respectively. Using word embeddings, each word in W, A_1 and A_2 can be mapped into a k-dimensional word vector $w \in R^k$. With a slight abuse of notation, we use $W, A_1 and A_2$ to denote the set of vectors rather than the set of words below. The scoring algorithm was executed as follows.

First, for each word list A_i (*i* = *1, 2*), the center a_i was computed using Equation (2), where the mid-point $m = (a_1 + a_2)/2$ was taken:

$$a_i = \frac{1}{|A_i|} \sum_{a \in A_i} a. \tag{2}$$

Second, to center the vectors, all vectors in $W$and A_i were shifted by $-m$, and the sets of centered vectors were denoted as $\hat{W} = W - m$ and $\hat{A_i} = A_i - m$, respectively:

$$\hat{W} = \{w - m: w \in W\},\ \hat{A_i}, = \{a - m: a \in A_i\}. \tag{3}$$

We apply the shifting method to both $A_1$and $A_2$and denote the results as $\hat{A_1} and \hat{A_2}$. For any $w \in \hat{W}$ and $a \in \hat{A_i}$, the matching score $m(w, a)$ is defined as follows:

$$m(w, a) = sign(d) \times \sigma_{p,r}(|d|), d = \frac{w \cdot a}{||w||_2 \cdot ||a||_2}; \tag{4}$$

$$\sigma_{p,r}(x) = \left[1 + \left(\frac{1 - h_p(x)}{h_p(x)}\right)^r\right]^{-1}, h_p(x) = \frac{2p-1}{2p(1-p)}x^2 + \frac{1-2p^2}{2p(1-p)}x; \tag{5}$$

where p (ranges between 0 and 1) and r (> 0) are the parameters for our method. The purpose of $\sigma_{p,r}(x), h_p(x), p$ and r ,are discussed further below. Then, for each $w \in \hat{W}$, the affinity score S toward words list $\hat{A_i}$ is denoted by the equation below:

$$S\left(w, \hat{A_i}\right) = \sum_{a \in \hat{A_i}} m(w, a). \tag{6}$$

Finally, the affinity score of a text W toward word list A_i is the sum of the scores for all vectors, calculated as follows:

$$S\left(\hat{W}, \hat{A_i}\right) = \sum_{w \in \hat{W}} S\left(w, \hat{A_i}\right). \tag{7}$$

Equations (2) and (3) reposition the vectors such that the contrast in word directions between the vectors in A_1andA_2 are distinguishable, making the algorithm more sensitive to the differentiation between, for example, masculine and feminine words. Equation (4) is based on cosine similarity scores but applies a sigmoid function to the scores such that values close to zero tend to be pushed toward zero and values close to ± 1 are amplified toward ± 1. The goal of Equation (3) is to reduce noise in cosine similarity. For $\sigma_{p,r}(x)$ in Equations (4) and (5), parameter r determines how rapidly the values approach the two ends (i.e., 0 and 1), and

parameter p determines the threshold above which the values are pushed toward 1. In simplified terms, [0, p) will be mapped toward 0, $(p, 1]$ will be mapped toward 1, and $\sigma_{p,r}(p) = 0.5$.

We used the 2017 version of the Global Vectors for Word Representation (GloVe)[56], which is one of the most prominent and widely-used word embedding tools in recent years. In GloVe, each word is represented by a 300-dimension vector. We set p to be 0.7 and r to be 5, which means a cosine similarity score of 0.7 is mapped toward 0.5, scores between –0.7 and 0.7 are mapped toward 0, and scores above 0.7 or below –0.7 are mapped toward 1 and –1, respectively. Robustness checks (Tables S4, S5) showed that using alternative p and r cut-offs would yield affinity scores that are closely correlated with those used in our analysis. Our scoring algorithm is further validated by comparing the word embedding results with a manually expert-labelled dataset based on a randomly selected sample of job ads.

The above computation is repeated for both A_1 and A_2 to obtain two affinity scores for each job ad for each dimension of gendered language, representing masculine and feminine gender orientations, respectively. Adjusting the parameters p and r, we attuned the scaling of the scores to be similar to the manual coding conducted by the team based on a randomly selected sample of job ads, such that the word embedding scores, $S(\hat{W}, \hat{A}_1)$ and $S(\hat{W}, \hat{A}_2)$, are akin to counting the total number of appearances of target and related words in a job ad, with words that are opposite in semantic meaning to the ones in our inventory taking negative values. The scoring algorithm was applied to single-word items in our inventory.

Table S4. Pearson's correlations between alternative *p* cut-offs (*p* = 0.65/0.75) for calculating word embeddings and the cut-off point (*p* = 0.70) used for the analysis reported in the main article

	A random week before COVID-19; *N* = 136,102 job ads (7 May 2018 to 13 May 2018)		A random week during COVID-19; *N* = 273,663 job ads (12 November 2021 to 18 November 2021)	
Dimension	*p* = 0.65	*p* = 0.75	*p* = 0.65	*p* = 0.75
Explicit gender references	0.892	0.692	0.767	0.645
Gendered psychological cues	0.950	0.984	0.952	0.984
Gendered work roles	0.836	0.957	0.851	0.964
Work-family cues	0.909	0.953	0.913	0.954
EDI policy	0.952	0.990	0.969	0.994
EDI culture	0.952	0.989	0.959	0.992

Note: EDI = Equality, diversity, and inclusion. In our main analysis, our word embedding algorithm used the cut-off point of 0.7 for the hyperparameter *p*. To ensure the robustness of our findings, we ran the same scoring algorithm on four randomly sampled datasets from both UK (random week) and Canada (random month), covering both pre-COVID and COVID periods, using alternative *p* values of 0.65 and 0.75. The hyperparameter *r* is fixed to be the same as in the main analysis (i.e., 5). The results in this table show that Pearson's correlation coefficients the gender/DEI language scores based on the alternative *p* values are highly correlated with those based on the *p* value used in our main analysis.

For multi-word phrases, we used the method of exact matching and counting (21, 25), with the appearance of each phrase taking a score of 1. This strategy was used because technically it is difficult for word embedding to accurately handle long phrases; substantively, the long phrases in our inventory are highly distinctive and have relatively low frequencies of

appearance in our dataset; and methodologically, words in job ads that exactly match words in our inventory would have been assigned a score of 1 by the word embedding scoring procedure. The exact-matching score for multi-word phrases is then added to the word embedding score for each job ad within each direction (i.e., feminine and masculine) for a given dimension (e.g., feminine psychological cues).

Table S5. Pearson's correlations between alternative *r* cut-offs (*r* = 4/6/10) for calculating word embeddings and the cut-off point (*r* = 5) used for the analysis reported in the main article

	A random week before COVID-19; *N* = 136,102 job ads (7 May 2018 to 13 May 2018)			A random week during COVID-19; *N* = 273,663 job ads (12 November 2021 to 18 November 2021)		
Dimension	*r* = 4	*r* = 6	*r* = 10	*r* = 4	*r* = 6	*r* = 10
Explicit gender references	0.876	0.855	0.628	0.814	0.873	0.713
Gendered psychological cues	0.996	0.998	0.989	0.996	0.998	0.989
Gendered work roles	0.935	0.985	0.952	0.940	0.987	0.959
Work-family cues	0.994	0.998	0.980	0.995	0.998	0.984
EDI policy	0.989	0.998	0.991	0.994	0.999	0.995
EDI culture	0.984	0.997	0.991	0.987	0.998	0.992

Note: DEI = diversity, equality, and inclusion. In our main analysis, our word embedding algorithm used the cut-off point of 5 for the hyperparameter *r*. To ensure the robustness of our findings, we ran the same scoring algorithm on four randomly sampled datasets from both UK (random week) and Canada (random month), covering both pre-COVID and COVID periods, using alternative *r* values of 4, 6, and 10. The hyperparameter *r* is fixed to be the same as in the main analysis (i.e., 0.7). The results in this table show that Pearson's correlation coefficients the gender and DEI scores based on the alternative *r* values are closely correlated with those based on the *r* value used in our main analysis.

To yield a single score for each dimension of gendered language for each job ad, we subtracted the score for the masculine direction from the score for the feminine direction, such that a lower score indicates a more masculine orientation and a higher score indicates a more feminine orientation. When only one direction is considered (i.e., EDI policy and EDI culture), the scores were directly computed by summing up the values of EDI-related vectors for each dimension within each job ad without executing Equations (2) and (3), with a higher score indicating a more pro-EDI orientation. To minimize the influence of outlier cases, we bottom- and top-coded the scores for each dimension at the 3rd and 97th percentiles (all results robust to alternative cut-offs such as the 1st and 99th percentiles). Finally, to facilitate data analysis and interpretation, we scaled the scores for each dimension to range from 0 to 100, with 0 indicating the most masculine/least pro-EDI job ad and 100 indicating the most feminine/pro-EDI ad.

We further validated our algorithm using the BIOS dataset (https://paperswithcode.com/dataset/biasbios), which includes personal biographies categorized by gender across various occupations, comprising a total of 255,710 samples. Given the limited availability of publicly available high-quality validation datasets, the BIOS dataset is particularly well-suited for our validation: the individual biographies, covering professional information such as occupational and career histories, are highly related and akin to labor market texts including job ads and job applications (e.g., resumes). To render our validation comparable with existing baselines, the validation drew on our explicit gender references by calculating the difference between femininity and masculinity scores, with designated gender serving as the ground truth label. Our algorithm achieved high performance, with an Area Under the ROC Curve (AUC) of

0.99 in classifying gender, as depicted in Figure S2 below. The AUC metric gauges the model's ability to accurately differentiate between classes—in this case, gender categories. The performance of our algorithm is comparable with two baseline methods discussed in the BOLD study (72), which utilize word embeddings' gender direction (i.e., she – he) to calculate gender polarity scores, thereby underscoring the efficacy and potential applicability of our scoring approach in broader natural language processing tasks.

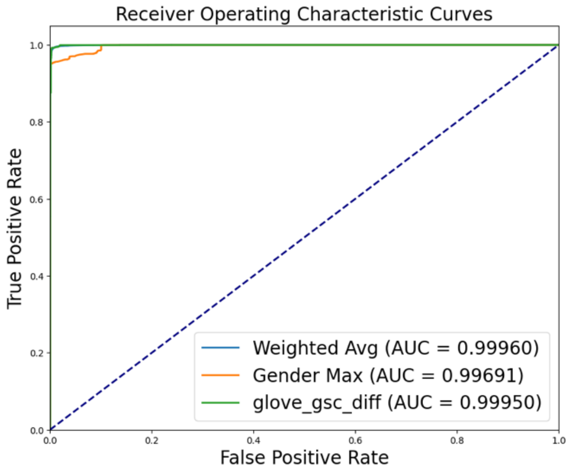

Fig. S2. AUC plot for algorithm validation and benchmarking

4.2. Descriptive statistics for gender/EDI language

Table S6 presents the descriptive statistics for each dimension of gender/EDI language use. Table S7 presents the mean gender/EDI language use scores for each of the 189 industry-occupation groups used in our analysis. Fig. S3 presents the standardized mean scores for each of the six dimensions of language across the same 189 industry-occupation groups, with a lighter color indicating more feminine/pro-EDI language. The results illustrate notable variations across industries and occupations. For example, job ads for electricity, gas, steam, and air conditioning tend to include a high level of explicitly masculine rather than feminine references. Feminine rather than masculine psychological and work-family cues tend to feature strongly in ads for care, leisure, and service occupations. Language describing masculine rather than feminine work roles is particularly prevalent in ads for process, plant, and machine occupations. EDI policy pledges are prevalent in job ads for international organizations, and language signaling workplace EDI culture tends to feature prominently in job ads for care, leisure, and service industries.

Table S6. Descriptive statistics for gender/EDI language

Job ad level (*N* = 28,609,485)	Mean/SD/Skewness/Kurtosis	Histogram (percentage)
Explicit gender references (high = feminine)	68.759/23.940/–1.085/3.816	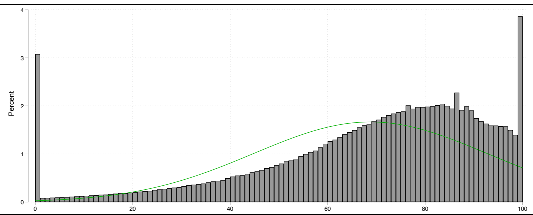

Gendered psychological cues (high = feminine)	57.859 /22.864/–0.603 /3.155	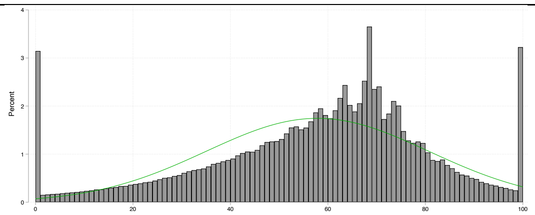
Gendered work roles (high = feminine)	72.842 /24.516/–1.292/4.193	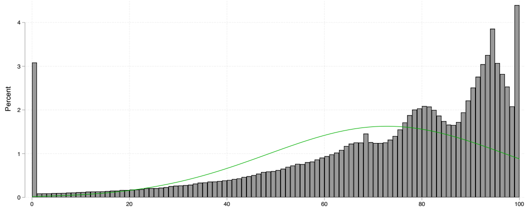
Work-family cues (high = family-friendly, feminine)	37.680/22.279/0.986/3.850	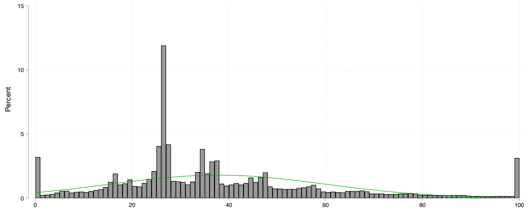
EDI policy (high = pro-EDI)	15.841/25.460/1.987/6.174	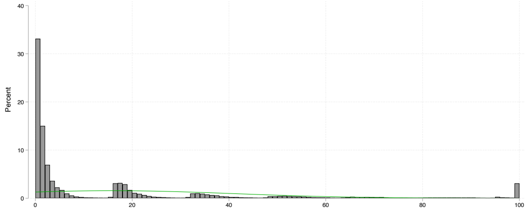
EDI culture (high = pro-EDI)	30.617/25.096/1.073/3.558	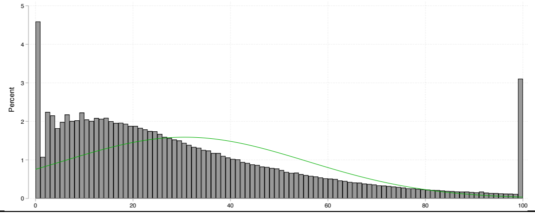

Table S7. Gender and EDI language in job ads across 189 industry-occupation groups, higher score = more feminine / pro-EDI (*N* = 28,609,485 job ads)

Industry (SIC level 1)	Occupation (SOC level1)								
	1	2	3	4	5	6	7	8	9
**Explicit gender references**									
A	67.63	68.70	72.32	72.15	76.07	72.63	67.61	78.67	83.16
B	63.44	64.34	63.90	63.75	74.14	66.14	69.58	76.37	70.09
C	71.19	72.62	74.60	74.88	78.83	67.54	71.88	78.59	76.21
D	58.37	58.62	59.92	60.07	63.73	61.97	62.70	63.75	57.30
E	67.13	68.73	66.24	69.45	69.63	69.80	67.71	76.71	73.39
F	68.55	69.87	71.13	71.18	81.93	65.44	67.91	80.70	75.96
G	67.04	66.49	66.99	68.59	74.55	65.59	64.62	64.89	67.27
H	64.93	66.85	67.70	64.04	71.80	67.34	66.08	81.08	74.33
I	69.82	68.31	68.06	67.68	71.44	66.24	68.02	74.39	69.58
J	60.15	64.57	63.79	66.21	69.28	65.89	62.40	73.48	66.94

K	60.96	62.74	64.38	67.45	64.55	63.05	60.56	65.10	65.06
L	70.78	69.14	69.80	70.94	71.13	64.53	68.06	74.20	73.00
M	61.41	69.70	69.09	70.30	70.49	67.84	65.82	73.26	67.94
N	68.29	69.36	69.20	70.18	73.87	70.25	68.57	75.72	76.51
O	61.28	60.52	62.51	64.24	67.55	66.65	61.45	68.23	68.25
P	65.72	69.79	66.49	68.36	67.66	70.01	66.08	72.83	71.54
Q	66.71	66.45	64.75	67.70	68.25	68.36	65.67	66.02	67.17
R	68.43	67.93	70.21	70.56	69.72	69.32	68.23	71.79	73.34
S	68.73	67.72	67.32	71.13	71.92	72.31	67.75	74.15	74.08
T	57.09	62.35	56.89	63.70	68.85	68.27	63.56	65.48	83.25
U	55.24	66.69	67.11	67.31	70.53	66.44	69.74	57.64	65.93
**Gendered psycho-logical cues**									
A	41.51	48.42	52.50	50.18	56.95	62.79	45.04	56.58	63.75
B	37.88	38.70	40.02	42.99	54.55	55.32	47.20	55.71	60.12
C	46.40	52.86	54.64	55.12	60.57	55.07	49.74	62.01	61.93
D	34.35	38.41	38.58	39.29	44.68	41.77	37.44	44.27	48.26
E	46.67	51.58	50.62	51.30	55.43	56.06	47.02	60.83	61.70
F	48.70	54.70	54.57	54.69	63.97	64.10	52.71	63.09	62.13
G	43.04	52.43	52.90	54.39	58.95	57.58	54.32	62.79	62.75
H	45.25	52.86	52.97	57.71	59.38	59.14	51.34	67.08	64.65
I	49.13	54.38	55.55	60.16	58.55	63.93	54.43	61.90	64.09
J	42.20	50.60	50.45	51.61	57.26	65.37	49.78	59.51	63.62
K	39.50	46.59	45.94	48.83	53.66	64.65	48.30	55.59	57.40
L	49.22	49.32	47.66	52.53	57.02	63.47	47.73	59.77	60.91
M	40.62	47.46	50.40	50.65	54.12	61.27	47.64	59.18	59.35
N	45.99	52.73	46.89	53.21	58.56	61.30	45.51	62.28	62.91
O	52.75	58.69	59.87	60.29	61.98	69.12	56.01	64.26	64.20
P	54.88	62.64	59.12	61.42	59.53	73.84	55.81	63.30	68.82
Q	55.84	67.18	64.84	66.12	62.02	69.12	60.82	66.47	69.07
R	48.44	53.28	55.07	55.28	54.97	55.12	47.42	56.02	58.15
S	50.55	55.33	55.42	58.24	58.06	62.25	52.91	62.42	63.61
T	52.52	58.15	57.39	60.34	58.57	67.28	53.67	67.89	70.36
U	40.48	46.51	49.30	54.23	49.30	63.77	46.02	38.46	44.61
**Gendered work roles**									
A	68.03	73.04	71.98	77.86	77.35	67.59	73.47	72.57	70.18
B	52.89	55.58	54.64	62.34	62.09	58.66	65.89	47.54	49.75
C	59.93	62.50	58.70	67.90	63.25	61.10	67.47	51.57	62.33
D	65.58	68.76	68.56	77.60	79.83	73.12	72.88	70.07	76.50
E	68.79	72.85	71.49	74.24	70.29	69.95	72.97	44.14	64.94
F	53.78	59.11	58.98	53.59	59.69	59.56	61.47	55.18	60.02
G	74.14	71.41	71.90	77.95	76.36	73.40	75.42	61.56	77.80

H	62.98	70.49	69.89	73.80	69.78	70.88	69.50	60.47	69.52
I	57.18	61.76	64.90	71.91	64.43	70.91	69.94	53.97	72.45
J	70.71	74.73	71.76	78.25	71.37	71.19	70.01	58.27	73.79
K	62.90	74.69	74.28	78.13	70.23	72.64	73.12	62.60	71.11
L	67.40	71.76	73.98	77.22	72.55	74.83	72.52	57.33	74.98
M	65.59	66.67	67.47	75.87	68.79	73.51	68.08	54.53	70.27
N	77.06	80.93	77.06	85.31	70.79	78.08	76.08	29.37	75.93
O	74.83	75.42	72.73	83.04	71.69	73.70	73.32	57.96	77.91
P	73.20	73.09	77.20	78.45	71.02	74.18	76.19	63.06	76.58
Q	72.05	73.47	72.44	80.48	72.62	78.76	72.54	49.47	75.19
R	64.97	70.58	64.14	76.95	72.75	74.43	66.90	47.47	85.56
S	57.84	62.81	65.93	78.36	71.02	71.26	60.04	47.94	65.07
T	68.03	73.04	71.98	77.86	77.35	67.59	73.47	72.57	70.18
U	52.89	55.58	54.64	62.34	62.09	58.66	65.89	47.54	49.75
**Work-family cues**									
A	35.16	34.97	34.09	40.09	33.17	42.96	35.44	29.91	31.89
B	32.67	29.31	30.59	32.49	30.97	41.47	40.38	31.09	35.23
C	35.59	33.96	35.19	36.77	32.08	38.03	37.58	34.11	37.38
D	40.86	42.04	43.33	42.94	41.22	41.14	38.95	41.72	42.14
E	37.10	37.08	39.11	41.09	34.80	38.92	39.76	33.25	37.59
F	37.85	37.06	36.67	39.42	29.30	42.84	37.76	30.90	36.08
G	38.22	40.03	38.53	40.80	34.77	44.80	41.60	40.70	44.50
H	36.58	34.94	35.40	43.93	33.97	33.96	37.57	28.42	37.30
I	38.86	37.11	41.25	44.60	39.91	46.27	44.16	40.64	48.93
J	36.87	35.28	35.88	37.19	33.86	43.32	40.85	32.77	42.64
K	37.19	37.70	38.83	38.86	41.52	49.73	40.94	39.81	44.47
L	35.05	37.27	36.34	36.97	36.31	46.60	39.04	34.88	40.44
M	35.26	35.39	35.78	37.41	36.68	45.86	37.05	35.67	41.05
N	35.26	37.16	38.63	38.14	35.03	36.96	38.09	34.19	42.80
O	38.35	40.17	37.99	38.10	35.25	37.07	37.66	35.61	39.53
P	34.73	36.86	35.01	34.35	31.03	37.99	34.47	31.01	36.26
Q	35.72	36.97	36.06	35.13	38.43	45.69	36.83	36.62	40.17
R	33.36	35.30	33.36	35.65	33.98	39.50	33.47	36.60	40.12
S	35.62	36.66	36.74	37.03	36.15	37.99	38.35	32.52	41.50
T	37.81	37.23	34.83	36.98	35.98	41.53	39.09	32.15	47.45
U	37.37	46.58	45.57	43.22	43.27	49.55	46.29	36.28	37.16
**EDI policy**									
A	13.98	17.14	14.79	14.50	11.48	11.70	18.56	9.08	6.04
B	31.68	35.64	34.73	29.07	16.29	30.30	22.55	15.65	17.90
C	9.77	10.06	8.44	9.09	5.09	14.73	10.20	6.30	7.85
D	33.32	31.74	30.48	29.26	26.42	23.67	19.32	25.45	30.26
E	11.73	13.74	15.04	13.31	12.10	14.55	11.31	8.69	12.42
F	17.03	19.95	16.75	17.56	7.54	26.15	13.29	7.94	12.92

G	12.46	14.89	13.19	10.66	5.66	15.19	13.22	10.70	10.53
H	17.12	16.81	15.64	22.26	14.17	18.25	15.67	5.87	11.68
I	9.35	13.58	12.88	14.75	8.16	16.03	11.39	5.65	9.61
J	23.80	23.16	21.39	19.38	12.08	18.90	18.96	13.02	11.86
K	31.18	27.59	24.36	21.12	19.28	21.85	20.77	20.61	18.81
L	12.88	16.79	17.13	15.74	13.27	23.14	16.28	14.69	15.96
M	20.16	14.03	16.89	13.65	12.07	18.52	15.16	12.81	13.07
N	16.20	15.94	11.71	14.26	8.88	12.44	14.93	9.50	9.78
O	29.01	30.75	28.01	28.08	22.32	26.41	25.90	20.74	29.22
P	22.06	16.98	21.40	21.52	14.39	16.10	19.55	7.25	16.32
Q	17.91	20.05	21.24	19.20	13.66	15.30	19.99	17.72	16.46
R	13.82	15.31	10.50	13.75	7.62	9.10	10.64	11.35	9.72
S	14.01	15.26	14.98	13.55	9.88	12.21	15.01	7.51	11.07
T	18.02	18.28	24.07	16.79	7.02	12.02	19.42	16.52	2.36
U	41.51	55.62	47.82	21.35	43.92	35.09	42.91	51.45	52.69
**EDI culture**									
A	28.94	28.60	25.80	24.70	18.63	31.47	27.18	19.02	16.30
B	37.55	36.39	36.59	34.39	20.07	38.39	29.29	18.12	24.88
C	25.91	23.24	21.74	20.51	14.59	35.60	23.57	14.06	17.71
D	37.98	35.67	36.18	35.71	24.92	35.39	28.33	30.34	33.72
E	24.73	24.96	28.15	25.90	21.61	25.79	25.21	14.94	18.87
F	28.54	27.96	26.44	26.55	9.90	45.43	26.20	11.51	17.68
G	31.59	32.88	28.75	25.39	17.22	34.79	27.64	21.88	24.44
H	31.19	29.13	29.05	25.67	21.75	33.86	27.99	10.13	18.21
I	26.46	28.15	29.57	28.15	22.89	31.97	28.62	18.04	24.60
J	39.42	32.01	35.04	31.70	25.14	43.12	32.14	21.68	26.17
K	38.59	35.02	33.05	29.89	31.15	58.09	32.26	28.90	31.28
L	23.48	26.37	26.40	25.30	21.90	47.30	25.30	18.83	21.92
M	39.07	26.85	27.97	27.37	24.45	39.21	29.58	21.08	25.85
N	30.08	28.36	27.71	26.98	18.89	32.34	27.10	16.62	18.31
O	37.65	38.07	37.06	33.72	25.69	38.42	36.54	23.99	24.74
P	32.43	24.58	31.93	30.16	27.16	29.89	31.74	20.27	24.79
Q	42.55	42.76	43.42	34.97	33.51	51.76	39.16	33.70	36.10
R	28.97	27.72	26.70	26.51	22.55	31.23	27.59	22.34	22.96
S	30.02	29.81	31.53	25.74	20.85	31.48	30.48	19.09	22.03
T	41.32	41.40	43.49	30.92	25.68	41.23	40.69	23.42	7.23
U	52.44	43.88	46.21	33.09	38.74	41.02	45.03	50.35	39.95

Note: All scores are scaled to range from 0 to 100. EDI = Equality, diversity, and inclusion. SIC = Standard industry classification 2007, where A = Agriculture, forestry and fishing, B = Mining and quarrying, C = Manufacturing, D = Electricity, gas, steam and air conditioning supply, E = Water supply, sewerage, waste management and remediation activities, F = Construction, G = Wholesale and retail trade; repair of motor vehicles and motorcycles, H = Transportation and storage, I = Accommodation and food service activities, J = Information and communication, K = Financial and insurance activities, L = Real estate activities, M = Professional, scientific and technical activities, N = Administrative and support service activities, O = Public administration and defence; compulsory social security, P = Education, Q =

Human health and social work activities, R = Arts, entertainment and recreation, S = Other service activities, T = Activities of households as employers; undifferentiated goods- and services-producing activities of households for own use, U = Activities of extraterritorial/international organisations and bodies. SOC = Standard occupation classification 2020, where 1= Managers, directors, and senior officials, 2 = Professional occupations, 3 = Associate professional and technical occupations, 4 = Administrative and secretarial occupations, 5 = Skilled trades occupations, 6 = Caring, leisure and other service occupations, 7 = Sales and customer service occupations, 8 = Process, plant, and machine operatives, 9 = Elementary occupations.

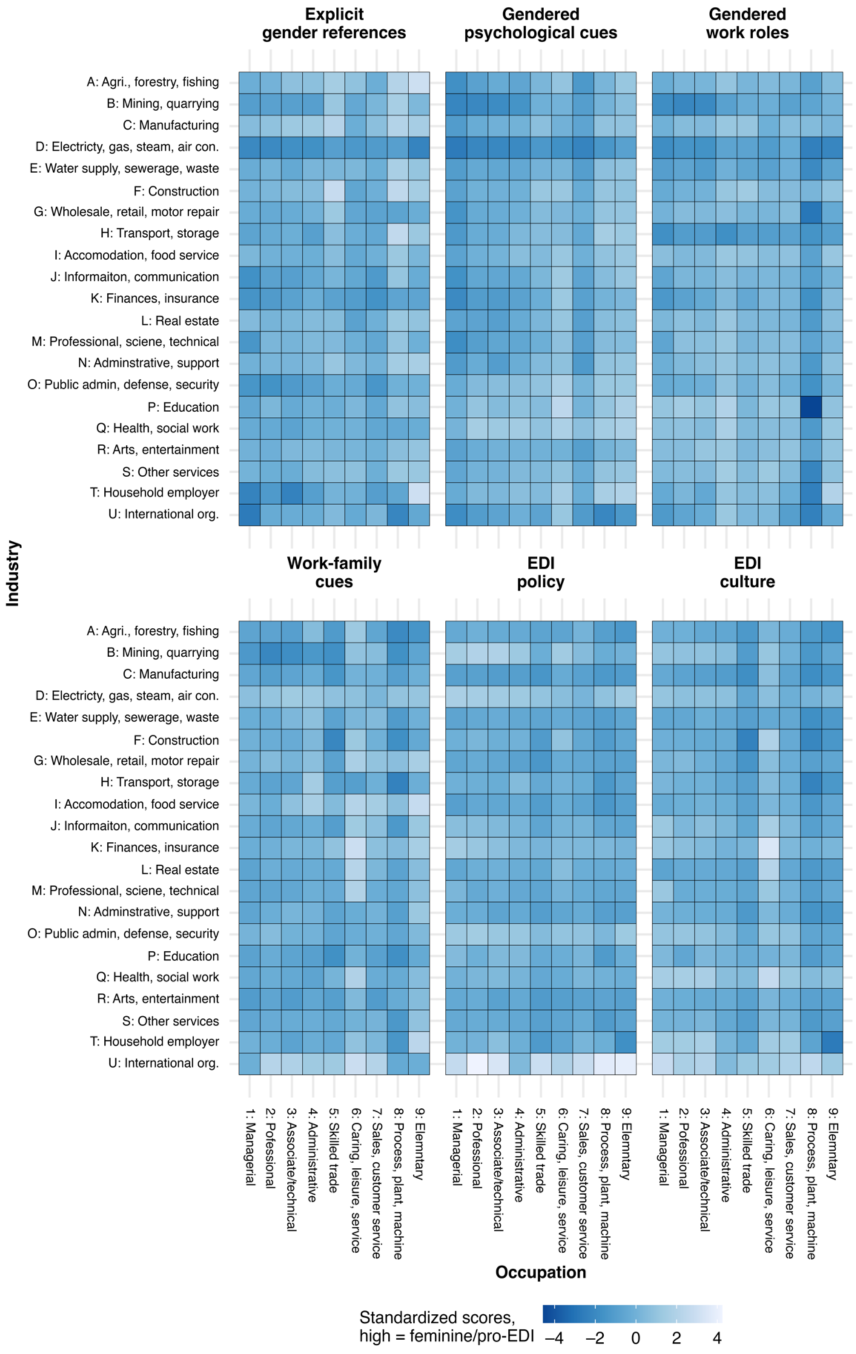

Fig. S3. Gender/EDI language in job ads across 189 industry-occupation groups. EDI = Equality, diversity, and inclusion. Calculated based on 28,609,485 UK job ads between January

2018 and June 2013. For presentation purposes, the scores were standardized within each dimension to take a mean of 0 and a standard deviation of 1. Industry is measured at level 1 of the Standard Industry Classification (SIC), and occupation is measured at level 1 of the Standard Occupation Classification (SOC).

Although we do not provide an exhaustive description and discussion of the distribution of the gender/EDI language scores here, our multidimensional inventory, along with the results presented in Table S6 and Fig. S3, provides a useful roadmap for organizations to systematically home in on specific aspects of language used in job ads.

Supplementary Materials 5: Instrumental variables

The relationship between language in job ads and labor force composition could be bidirectional and thus endogenous. As reported in *Supplementary Materials 7*, the Hausman tests confirmed the presence of endogeneity in the relationship between many dimensions of language in job ads and labor force gender/racial composition (73). In this case, we adopted an IV approach to mitigate endogeneity and help disentangle the bidirectional influences. We carefully chose the IVs based on the three core IV assumptions (73): (a) the IV is associated with the endogenous predictor ("relevance"); (b) it only affects the outcome through the instrumented predictor ("exclusion restriction"); and (c) it is uncorrelated with the error term of the outcome ("independence"). We discuss our IVs in relation to each of the assumptions, as well as the tests we conducted to support their validity. The validity of our IVs is grounded in careful theoretical considerations; and our confidence in the validity of the IVs is further bolstered by additional statistical explorations. We present the descriptive statistics for the IVs in this section below and the detailed test results for the IVs as a part of the model results in *Supplementary Materials 7*.

In estimating the impact of language in job ads on labor force composition, we used the word count of each job ad and its squared term as instruments for each dimension of gender/EDI language in job ads. The distribution of the IV is shown in Table S8.

Table S8. Descriptive statistics for job ad word count

**Job ad level (*N* = 28,609,485)**	**Mean/SD/Skewness/Kurtosis**	**Histogram (percentage)**
Job ad word count	447.969/246.525/0.795/3.171	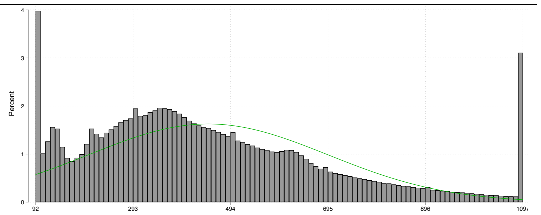

As longer job ads contain more words/phrases, there is good reason to expect that the length of job ads strongly predicts the number of linguistic cues in the ads (relevance). There is little reason to expect the impact of gender/EDI language in job ads on labor force composition to differ between shorter and longer job ads over and above the amount of linguistic cues they contain, which is already captured by the gender/EDI language scores. The under-identification tests show that the IVs are relevant, as the Kleibergen-Paap rk LM (Langrange multiplier) statistics rejected the null hypothesis that the IVs are irrelevant ($P < 0.001$ for all models); and the Kleibergen-Paap rk Wald *F* weak-IV tests further show that the IVs strongly identified the endogenous predictors ($F > 10$, $P < 0.001$ for all models)[63]. Although it is possible in theory that labor forces with a greater minority representation may be more likely to include additional information such as EDI statements in job ads that may make the ads longer or vice versa, our tests showed that job ad word count bears hardly any association with labor force gender (Pearson's $r = 0.063$) or racial (Pearson's $r = 0.043$) composition. These results give us confidence that the length of job ads does not vary systematically across industry-occupation groups, and the distribution of the length of job ads across industry-occupation groups

characterized by differential labor force gender/racial composition is largely random. Nor is there any theory or prior research suggesting that the length of job ads directly shapes labor force composition (exclusion). The Sargan-Hansen over-identification test examines the joint null hypothesis that the IVs are uncorrelated with the error term of the outcome variable (independence) and that the excluded IVs are correctly excluded from the equation (73). Across all models, the Sargan-Hansen test results cannot reject this null hypothesis (*P* > 0.05 for all models).

In estimating the impact of labor force gender/racial composition on gender/EDI language in job ads, we used historical labor force gender/racial/migrant composition measures at the first levels of SIC and SOC (not their interaction) as instruments for the current gender/racial composition across the 189 industry-occupation groups (the interaction between the first levels of SIC and SOC), with a long 20-year lag. The IVs captured the proportion of the labor force in major industries/occupations composed of women as opposed to men, racial minority as opposed to white workers, and migrants born outside the UK as opposed to UK-born workers, respectively, in 2001–2002. For the models estimating the non-linear impact of labor force composition on language in job ads, we included the quadratic, in addition to linear, terms of the historical labor force composition measures as IVs. The distributions of these IVs are presented in Table S9.

Table S9. Descriptive statistics for lagged labor force gender/racial composition

	**Mean/SD/Skewness/ Kurtosis**	**Histogram (percentage)**
**Industry SIC level 1 (*N* = 21)**		
% women in labor force in 2001–2002	42.728/19.498/ 0.118/2.160	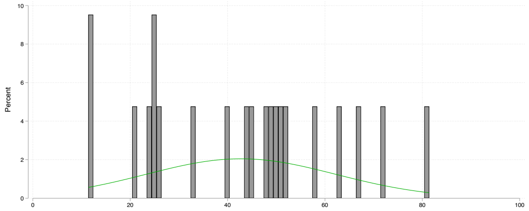
% racial minority in labor force in 2001–2002	5.939/3.487/1.520/5.976	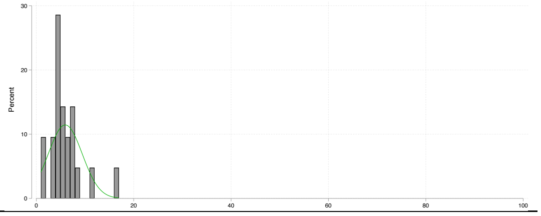
**Occupation SOC level 1 (*N* = 9)**		
% women in labor force in 2001–2002	47.292/26.724/0.041/1.817	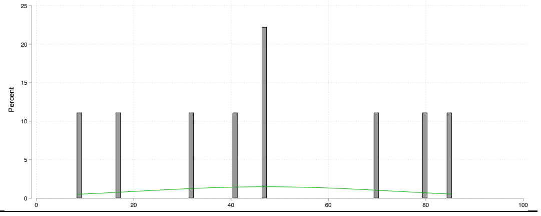

% racial minority in labor force in 2001–2002	6.112/1.155/–0.671/2.884	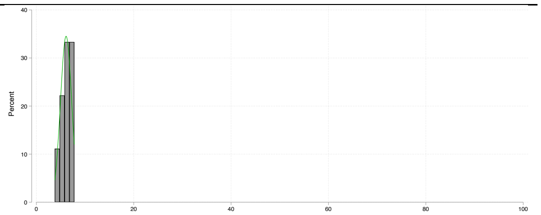
% migrant in labor force in 2001–2002	4.513/1.169/0.241/1.686	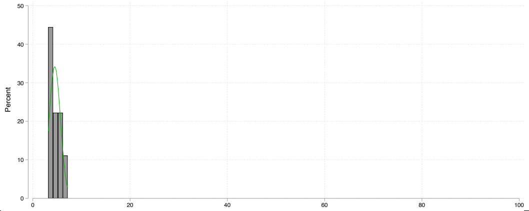

In terms of the IV assumptions, current industry-occupation force gender/racial composition has evolved from and would thus be associated with historical labor force composition (relevance). The under-identification tests show that the IVs are relevant, as the Anderson canonical correlations tests rejected the null hypothesis that the IVs are irrelevant ($P < 0.001$ for all models), and the Cragg-Donald Wald *F* weak-IV tests further show that the IVs strongly identified the endogenous predictors ($F > 10$, $P < 0.001$ for all models). It might be possible that the historical workforces crafted job ads in given ways based on or in response to their composition, and the legacy job ads are reused for drafting current job ads—a possibility that could violate the exclusion assumption. However, we have deliberately chosen a long (two-decade lag) for our IVs such that this possibility is extremely unlikely. In the ~20 years between when the lagged labor force composition measures were taken (2001–2002) and the focal period of our study (2018–2023), a number of drastic social changes took place that render it extremely unlikely and indeed infeasible for employers and recruitment agencies to recycle legacy job ads in current job advertising.

First, the UK labor market, employers, and job roles have all undergone substantial changes in the 20 years, making it very difficult, impractical, and unbeneficial to recycle job ads from 20 years ago. Indeed, employers, recruiters, and HR professionals draft ads for substantially reconfigured job roles, person specifications, and labor market contexts today, while much of the specific historical job descriptions are out of not suitable for today's job roles and labor market and legal contexts. Second, a number of conceptual dimensions captured in our word inventory were mainstreamed into the labor market, and public discourse only in the past few years; these dimensions hardly featured in the 2001–2002 labor market when our lagged labor force composition IVs were taken. For example, work-family balance and flexible work have only recently been mainstreamed into job configurations and related job ads. Third, the UK's Equality Act 2010, passed and implemented after our lagged IVs, has substantially changed legal expectations and regulations regarding equality language use in public discourse, including job ads. Fourth, recent movements such as #metoo and #BlackLivesMatter and a global diffusion of ideals pertaining to equality, diversity, and inclusion (EDI) have created new norms and practices in the workplace and labor markets. Again, the mainstreaming of EDI into labor market processes and standards was only a recent development that came way after our lagged IVs. Finally, rapid digitalization and the proliferation of job advertising platforms have set new standards and procedures for, and have thus drastically changed, how jobs are advertised

and how the ads are written. While job advertising in the early 2000s (i.e., the early stage of mass digitalization) relied heavily on print media with job ads framed as short snippets in, for example, newspapers and magazines, mass digitalization of job advertising and hiring in the past decade or so has drastically changed how job ads are produced and presented.

Considering these developments, we have good reasons to believe that the current labor force is extremely unlikely to recycle legacy job ads developed by the historical labor force captured by our lagged IVs. Rather, there is an imperative for the current labor force to stay agile and be sensitive to rapidly changing labor market regulations, configurations, and work roles in strategizing, drafting, and tailoring the job ads analyzed in our study. Because it is the current labor force that is responsible for writing the job ads and given drastic labor market changes that render it unlikely for legacy job ads 20 years ago to be reused today, we expect historical labor force composition to relate to language in job ads only indirectly through current labor force composition (exclusion). Across all models, none of the Sargan-Hansen over-identification statistics was statistically significant at the 5% level, so we cannot reject the joint null hypothesis that the IVs are uncorrelated with the error term of the outcome variable (independence) and that the excluded IVs are correctly excluded from the equation (73). Early in our research, we also tested longer and shorter lags for the IVs: (a) although historical labor force participation with longer (than 20-year) lags make adequate IVs, they less strongly identify the models compared to our 20-year lag; in this case, our 20-year lag is preferred; (b) shorter lags, particularly with labor force composition measures taken after the mass digitalization of labor market processes (for example, ca. 2010 – a 10-year lag), did not pass the Sargan-Hansen tests, as the error terms of these shorter lagged IVs were correlated with the errors of the equation; this is as we expected according to theory in that unlike our 20-year lagged measures predating and exogenous to the mass digitalization of job advertising, the shorter lags may be vulnerable to potential endogeneity.

Supplementary Materials 6: Modeling strategy and control variables

To prepare our data for modeling, we merged the job ad and labor force composition datasets by industry (SIC 2007 level 1, 21 categories), occupation (SOC 2010 level 1, 9 categories), and cross-tabulated industry-occupation groups (189 categories). Using the merged dataset, we fitted our models in two steps.

First, we examined the impact of gender/EDI language in job ads on labor force gender/racial composition across 189 industry-occupation groups. To mitigate endogeneity and reverse causality, we estimated two-stage IV generalized method of moments (GMM) regression models. The first-stage models used the IVs introduced in the previous section to predict each dimension of gender/EDI language across the 28.6 million job ads, and the second-stage models used the predicted gender/EDI language scores from the first stage for the 28.6 million job ads to predict labor force gender/racial composition across the 189 industry-occupation groups. The two stages were jointly estimated using the *ivreg2* package in Stata 18 to obtain correct standard errors (74). Because the job ads are nested within the 189 industry-occupation groups, we estimated clustered standard errors to account for the data structure (75). The GMM, rather than the conventional least squares method, was used because of its estimation efficiency and accuracy (74). For each dimension of labor force composition (gender and race), we modeled each dimension of gender/EDI language in separate models (a) to estimate their unconstrained impact on labor force composition, and (b) to ensure the IVs clearly and adequately identified the endogenous predictors, including having a larger number of IVs than the endogenous predictors for the Sargan-Hansen over-identification test (73, 74). We did not examine non-linearity in the impact of gender/EDI language on labor force composition, because there was no compelling theoretical reason to expect the impact to be non-linear. We controlled for three variables in all models, which may confound the relationship between language in job ads and labor force composition: (a) year of job ads (2018–2023), (b) region of job ads (covering 10 broad regions across the UK), and (c) the source of job ads (covering 7 channels of job advertising, e.g., recruiter websites, aggregator sites, etc.). Detailed descriptive statistics for the control variables are presented in Table S10 below. Early in this research, we also experimented with controlling for the month of job ads as well as the interaction between month and year. However, because the inclusion of these variables did not affect the results for our focal predictors and contributed little to improving the overall model fit, they were excluded from our final analysis.

Table S10. Descriptive statistics for control variables

**Job ad level (*N* = 28,609,485)**	**Proportion**	**Histogram (percentage)**
Year of job ads	2018 = 0.16<br>2019 = 0.14<br>2020 = 0.13<br>2021 = 0.19<br>2022 = 0.26<br>2023 = 0.12	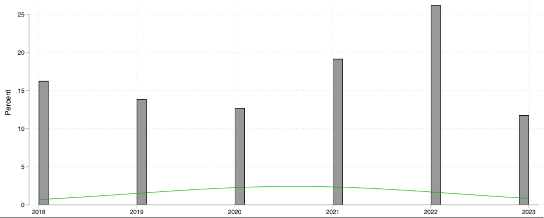

Region of job ads	1 (East Midlands) = 0.06<br>2 (East of England) = 0.08<br>3 (Greater London) = 0.15<br>4 (North East) = 0.02<br>5 (North West) = 0.10<br>6 (South East) = 0.14<br>7 (South West) = 0.08<br>8 (West Midlands) = 0.09<br>9 (Yorkshire and Humber) = 0.07<br>10 (Other, such as Scotland and Wales) = 0.21	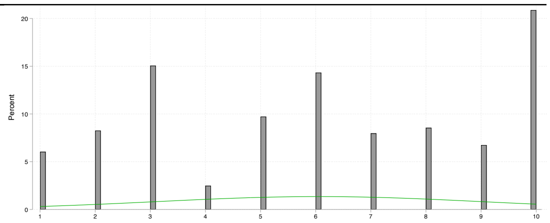
Source of job ads	1 (Employer website) = 0.09<br>2 (Education institutions) = 0.01<br>3 (Government website) = 0.01<br>4 (Job board) = 0.52<br>5 (Job intermediary/aggregator) = 0.27<br>6 (Recruiter) = 0.03<br>7 (Other) = 0.08	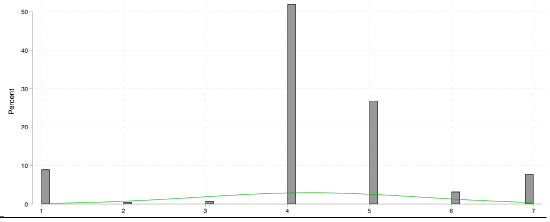

Next, we examined the impact of labor force gender/racial composition on each dimension of gender/EDI language in job ads. Similarly, we estimated two-stage IV GMM regression models. Because both the IVs and endogenous predictors were measured at the industry-occupation or industry/occupation level, it makes little sense to estimate the first-stage models based on the sample of 28.6 million job ads where the labor force composition values are assigned to individual job ads, with multiple duplicating records included in the sample for both the independent and dependent variables. In this case, we estimated the models based on the reduced sample of 189 industry-occupation groups, and calculated the dependent variables as the adjusted mean scores of each dimension of gender/EDI language for each of the 189 industry-occupation groups. The adjustments took account of the year, region, and source of job ads using the measures reported in Table S10. In the first-stage models, we regressed the current labor force gender/racial composition for the 189 industry-occupation groups on the IVs. In the second-stage models, we regressed the adjusted mean values of each dimension of gender/EDI language for the 189 industry-occupation groups on the predicted labor force gender/racial composition obtained from the first-stage models. We modeled the impact of labor force gender and racial composition on each dimension of gender/EDI language separately to understand their unconstrained impacts. Moreover, to test the compensation hypotheses (i.e., non-linearity), we included the quadratic, in addition to the linear, term of labor force gender/racial composition as an endogenous predictor in models (and accordingly included the quadratic terms of the IVs in the first-stage models).

Notably, we have conducted several supplementary analyses to ensure the robustness of our results. First, adjusting *p* values for multiple hypothesis testing does not alter our substantive conclusions (*Supplementary Materials 7, Tables S11–13*). Second, although we used the proportion of non-white workers to measure labor force racial composition in our main analysis, our further robustness checks using the alternative Blau diversity index (20) based on multiple ethnic/racial categories yielded substantively consistent results (*Supplementary Materials 8, Table S14*). Third, our study covers the 2018–2023 period, and our further analysis considered potential heterogeneities between 2018–2020 and 2021–2023 (e.g., COVID-19 and Black Lives Matter). We have taken 2021 rather than 2020 as the cut-off year because it is

plausible to expect any impact of COVID-19 and movements such as Black Lives Matter to take some time to result in changes in language used in job ads. The supplementary results show that our main findings remain substantively consistent across the time periods, and they further show that, as expected due to movements such as Black Lives Matter, the role of workforce racial composition in predicting EDI language in job ads is stronger in the latter period (*Supplementary Materials 8, Tables S15–S17*). Finally, although we do not have a strong theoretical motivation to hypothesize how gender language may affect labor force racial composition, we estimated supplementary models to examine the role of gender language in predicting racial minority representation across industry-occupation groups. The results show that none of the four dimensions of gender language plays a statistically significant role in predicting labor force racial composition (*Supplementary Materials 8, Tables S18*).

Supplementary Materials 7: Model results for the figures presented in the main article

Table S11. Two-stage instrumental-variable GMM regression models estimating the impact of gender/EDI language in job ads on labor force composition (for Fig. 2 in the main article)

	% women						% racial minority	
	B (*SE*) [*p*] {*q*}	*B* (*SE*) [*p*] {*q*}	*B* (*SE*) [*p*] {*q*}	*B* (*SE*) [*p*] {*q*}	*B* (*SE*) [*p*] {*q*}	*B* (*SE*) [*p*] {*q*}	*B* (*SE*) [*p*] {*q*}	*B* (*SE*) [*p*] {*q*}
Explicit gender references (high = feminine)	–0.074 (0.035) [0.034] {0.060}							
Gendered psychological cues (high = feminine)		–0.260 (0.125) [0.037] {0.060}						
Gendered work roles (high = feminine)			–0.096 (0.046) [0.037] {0.060}					
Work-family cues (high = feminine)				0.313 (0.141) [0.026] {0.060}				
EDI policy (high = pro-EDI)					0.102 (0.053) [0.052] {0.070}		0.005 (0.015) [0.715] {0.819}	
EDI culture (high = pro-EDI)						0.072 (0.034) [0.034] {0.060}		0.001 (0.009) [0.870] {0.871}
Constant	52.009 (3.472) [< 0.001]	60.261 (7.027) [< 0.001]	53.875 (4.097) [< 0.001]	34.314 (7.134) [< 0.001]	46.080 (3.418) [< 0.001]	44.928 (3.551) [< 0.001]	13.176 (0.685) [< 0.001]	13.235 (0.736) [< 0.001]
Control variables	Year, region, and source of job ads	Year, region, and source of job ads	Year, region, and source of job ads	Year, region, and source of job ads	Year, region, and source of job ads	Year, region, and source of job ads	Year, region, and source of job ads	Year, region, and source of job ads
Instrumental variables	Job ad word count – linear and quadratic terms	Job ad word count – linear and quadratic terms	Job ad word count – linear and quadratic terms	Job ad word count – linear and quadratic terms	Job ad word count – linear and quadratic terms	Job ad word count – linear and quadratic terms	Job ad word count – linear and quadratic terms	Job ad word count – linear and quadratic terms
Hausman endogeneity test, χ^2	0.403 [0.525]	11.972 [< 0.001]	11.479 [< 0.001]	2.655 [0.103]	0.961 [0.327]	9.443 [0.002]	0.000 [0.984]	1.788 [0.181]

Under-identification (Kleibergen-Paap rk LM statistic, χ^2)	42.398	49.052	47.545	42.616	39.864	32.108	39.864	32.108
	[< 0.001]	[< 0.001]	[< 0.001]	[< 0.001]	[< 0.001]	[< 0.001]	[< 0.001]	[< 0.001]
Weak identification (Kleibergen-Paap rk Wald *F* statistic)	40,655.09	51.878	659.266	331.7673	2,545.717	1,012.886	2,545.717	1,012.886
	[< 0.001]	[< 0.001]	[< 0.001]	[< 0.001]	[< 0.001]	[< 0.001]	[< 0.001]	[< 0.001]
Over-identification (Hansen J statistic, χ^2)	0.687	0.010	0.360	0.071	1.029	0.426	1.794	1.789
	[0.407]	[0.920]	[0.549]	[0.790]	[0.310]	[0.514]	[0.181]	[0.181]

Note: N = 28,609,485 job ads for all models. SE = Standard errors clustered at the level of industry-occupation groups (189 groups). GMM = Generalized method of moments. EDI = Equality, diversity, and inclusion. LM = Lagrange multiplier. First-stage model results are not shown. Racial minority refers to all ethnic groups other than white British, white Irish, and other white groups. {*q*} refer to Benjamini and Hochberg *q*-values adjusting for multiple hypothesis testing as described in Anderson (2008) (76).

Table S12. Two-stage instrumental-variable GMM regression models estimating the *linear* impact of labor force composition on gender/EDI language in job ads (all language scores adjusted for the year, region, and source of job ads; for Fig. 3 Panel A in the main article)

	Explicit gender references	Gendered psychological cues	Gendered work roles	Work-family cues	EDI policy	EDI culture	EDI policy	EDI culture
	B (*SE*) [*p*] {*q*}	*B* (*SE*) [*p*] {*q*}	*B* (*SE*) [*p*] {*q*}	*B* (*SE*) [*p*] {*q*}	*B* (*SE*) [*p*] {*q*}	*B* (*SE*) [*p*] {*q*}	*B* (*SE*) [*p*] {*q*}	*B* (*SE*) [*p*] {*q*}
% women	–0.056	0.283	0.197	0.084	–0.043	0.165		
	(0.015)	(0.061)	(0.024)	(0.012)	(0.049)	(0.025)		
	[< 0.001]	[< 0.001]	[< 0.001]	[< 0.001]	[0.384]	[< 0.001]		
	{0.001}	{0.001}	{0.001}	{0.001}	{0.384}	{0.001}		
% racial minority							0.765	0.806
							(0.163)	(0.168)
							[< 0.001]	[< 0.001]
							{0.001}	{0.001}
Constant	71.730	43.068	60.781	33.729	18.419	21.054	7.684	18.698
	(0.709)	(2.689)	(1.127)	(0.560)	(2.185)	(1.172)	(1.995)	(2.045)
	[< 0.001]	[< 0.001]	[< 0.001]	[< 0.001]	[0.011]	[< 0.001]	[< 0.001]	[< 0.001]
Instrumental variables	% women at SOC level 1 and SIC level 1 in 2001–2002	% migrants at SOC level 1 and % women at SIC level 1 in 2001–2002	% women at SOC level 1 and SIC level 1 in 2001–2002	% women at SOC level 1 and SIC level 1 in 2001–2002	% racial minority at SOC level 1 and % women at SIC level 1 in 2001–2002	% women at SOC level 1 and SIC level 1 in 2001–2002	% racial minority at SOC level 1 and SIC level 1 in 2001–2002	% migrants at SOC level 1 and % racial minority SIC level 1 in 2001–2002
Endogeneity test (Hausman test, χ^2)	5.120	25.348	0.010	22.245	0.739	17.005	21.696	15.031
	[0.024]	[< 0.001]	[0.921]	[< 0.001]	[0.390]	[< 0.001]	[< 0.001]	[< 0.001]
Under-identification test (Anderson's canonical correlations test, χ^2)	149.407	39.239	149.407	149.407	45.374	149.407	68.001	56.562
	[< 0.001]	[< 0.001]	[< 0.001]	[< 0.001]	[< 0.001]	[< 0.001]	[< 0.001]	[< 0.001]
Weak identification test (Cragg-Donald Wald *F* statistic)	350.937	24.367	350.937	350.937	29.380	350.937	52.278	39.719
	[< 0.001]	[< 0.001]	[< 0.001]	[< 0.001]	[< 0.001]	[< 0.001]	[< 0.001]	[< 0.001]
Over-identification test (Hansen J statistic, χ^2)	0.613	0.294	1.398	0.311	1.928	0.150	2.411	2.459
	[0.434]	[0.588]	[0.237]	[0.577]	[0.165]	[0.699]	[0.121]	[0.117]

Note: *N* = 189 industry-occupation groups for all models. SE = Standard errors. GMM = Generalized method of moments. EDI = Equality, diversity, and inclusion. LM = Lagrange multiplier. First-stage model results are not shown. Racial minority refers to all ethnic groups other than white British, white Irish, and other white groups. {*q*} refer to Benjamini and Hochberg *q*-values adjusting for multiple hypothesis testing as described in Anderson (2008) (76).

Table S13. Two-stage instrumental-variable GMM regression models estimating the *non-linear* impact of labor force composition on gender/EDI language in job ads (all language scores adjusted for the year, region, and source of job ads; for Fig. 3 Panel B in the main article)

	Explicit gender references	Gendered psychological cues	Gendered work roles	Work-family cues	EDI policy	EDI culture	EDI policy	EDI culture
	B (*SE*) [*p*] {*q*}	*B* (*SE*) [*p*] {*q*}	*B* (*SE*) [*p*] {*q*}	*B* (*SE*) [*p*] {*q*}	*B* (*SE*) [*p*] {*q*}	*B* (*SE*) [*p*] {*q*}	*B* (*SE*) [*p*] {*q*}	*B* (*SE*) [*p*] {*q*}
% women	–0.270	–0.051	0.181	0.440	0.329	0.196		
	(0.087)	(0.215)	(0.143)	(0.093)	(0.167)	(0.148)		
	[0.002]	[0.814]	[0.206]	[< 0.001]	[0.050]	[0.188]		
	{0.007}	{0.904}	{0.277}	{0.001}	{0.010}	{0.274}		
% women (squared)	0.002	0.004	0.000	–0.004	–0.004	–0.000		
	(0.001)	(0.002)	(0.002)	(0.001)	(0.002)	(0.002)		
	[0.014]	[0.098]	[0.928]	[< 0.001]	[0.062]	[0.847]		
	{0.036}	{0.157}	{0.928}	{0.001}	{0.110}	{0.904}		
% racial minority							–2.830	–0.366
							(0.684)	(0.429)
							[< 0.001]	[0.396]
							{0.001}	{0.488}
% racial minority (squared)							0.126	0.037
							(0.026)	(0.015)
							[< 0.001]	[0.015]
							{0.001}	{0.036}
Constant	74.890	47.957	61.101	28.751	11.224	20.525	27.257	26.574
	(1.421)	(4.110)	(2.344)	(1.776)	(2.739)	(2.432)	(3.986)	(2.975)
	[< 0.001]	[< 0.001]	[< 0.001]	[< 0.001]	[< 0.001]	[< 0.001]	[< 0.001]	[< 0.001]
Instrumental variables	% women SOC level 1 – linear and quadratic terms, % women SIC level 1 – linear and quadratic terms	% migrants SOC level 1 – linear and quadratic terms, % women SIC level 1 – linear and quadratic terms	% women SOC level 1 – linear and quadratic terms, % women SIC level 1 – linear and quadratic terms	% migrant SOC level 1 – linear and quadratic terms, % women SIC level 1 – linear and quadratic terms	% women SOC level 1 – linear and quadratic terms, % women SIC level 1 – linear and quadratic terms	% women SOC level 1 – linear and quadratic terms, % women SIC level 1 – linear and quadratic terms	% racial minority SOC level 1 – linear and quadratic terms, % racial minority SIC level 1 – linear and quadratic terms	% migrants SIC level 1 – linear and quadratic terms, % racial minority SIC level 1 – linear and quadratic terms
Endogeneity test (Hausman test, χ^2)	4.811	31.911	0.620	18.724	8.097	18.956	60.514	12.476
	[0.092]	[< 0.001]	[0.733]	[< 0.001]	[0.017]	[< 0.001]	[< 0.001]	[0.002]
Under-identification test (Anderson's canonical correlations test, χ^2)	54.056	40.108	54.056	40.108	54.056	54.056	36.939	45.733
	[< 0.001]	[< 0.001]	[< 0.001]	[< 0.001]	[< 0.001]	[< 0.001]	[< 0.001]	[< 0.001]

Weak identification test (Cragg-Donald Wald *F* statistic)	18.427	12.391	18.427	12.391	18.427	18.427	11.175	14.684
	[< 0.001]	[< 0.01]	[< 0.001]	[< 0.01]	[< 0.001]	[< 0.001]	[< 0.01]	[< 0.001]
Over-identification test (Hansen J statistic, χ^2)	1.597	1.304	3.493	4.654	4.230	1.520	2.150	5.685
	[0.450]	[0.521]	[0.174]	[0.097]	[0.121]	[0.468]	[0.341]	[0.058]

Note: *N* = 189 industry-occupation groups for all models. SE = Standard errors. GMM = Generalized method of moments. EDI = Equality, diversity, and inclusion. LM = Lagrange multiplier. First-stage model results are not shown. Racial minority refers to all ethnic groups other than white British, white Irish, and other white groups. {*q*} refer to Benjamini and Hochberg *q*-values adjusting for multiple hypothesis testing as described in Anderson (2008) (76).

Supplementary Materials 8: Supplementary analyses

Table S14. Two-stage instrumental-variable GMM regression models, measuring labor force racial composition using Blau diversity index

	Blau index measuring racial/ethnic diversity	Blau index measuring racial/ethnic diversity	EDI policy	EDI culture	EDI policy	EDI culture
	B (*SE*) [*p*]	*B* (*SE*) [*p*]	*B* (*SE*) [*p*]	*B* (*SE*) [*p*]	*B* (*SE*) [*p*]	*B* (*SE*) [*p*]
EDI policy	0.020					
	(0.023)					
	[0.370]					
EDI culture		0.010				
		(0.015)				
		[0.483]				
Blau index			0.463	0.508	–2.282	–0.732
			(0.103)	(0.107)	(0.529)	(0.374)
			[< 0.001]	[< 0.001]	[< 0.001]	[0.052]
Blau index (squared)					0.060	0.027
					(0.013)	(0.009)
					[< 0.001]	[0.003]
Constant	23.230	23.127	6.973	17.523	31.713	28.819
	(1.049)	(1.101)	(2.234)	(2.303)	(4.824)	(3.496)
	[< 0.001]	[< 0.001]	[< 0.001]	[< 0.001]	[< 0.001]	[< 0.001]
Instrumental variables	Job ad word count – linear and quadratic terms	Job ad word count – linear and quadratic terms	Blau index for ethnic diversity at SOC level 1 and SIC level 1 in 2001–2002	Blau index for ethnic diversity at SIC level 1 in 2001–2002, % migrants at SOC level 1 in 2001–2002	Blau index for ethnic diversity at SOC level 1 and SIC level 1 –linear and quadratic – in 2001–2002	Blau index for ethnic diversity at SIC level 1 – linear and quadratic – in 2001–2002, % women at SIC level 1 – linear and quadratic – in 2001–2002
N	28,609,485 job ads	28,609,485 job ads	189 industry-occupation groups	189 industry-occupation groups	189 industry-occupation groups	189 industry-occupation groups

Note: GMM = Generalized method of moments. EDI = Equality, diversity, and inclusion. LM = Lagrange multiplier. SE = Standard errors clustered at the level of industry-occupation groups (189 groups) for the first two models. First-stage model results not shown. Blau index measures the probability that two randomly selected individuals belong to different ethnic groups. The original Blau index ranges from 0 to 1, with a higher score indicates greater racial diversity in the labor force composition. For the ease of interpretation and comparison with our main results, we rescaled the measure to range from 0 to 100. We calculated Blau index for current labor force composition across the 189 industry-occupation groups based on 11 ethnic categories, as captured by the 2018–2023 Labor Force Survey: white British, white Irish, other white, mixed, Indian, Pakistani, Bangladeshi, Chinese, other Asian, Black African/Caribbean/Black/British, and other; and we calculated Blau index for the IVs (historical labor force ethnic composition) based on six ethnic categories, as the 2001–2002 Labor Force Survey did not capture more detailed ethnic groups: white, mixed, Asian, Black, Chinese, and other. Control variables for the first two models include the region, source and year of job ads. All instrumental variables have passed all the tests reported in *Supplementary Materials 7* as valid instruments.

Table S15. Two-stage instrumental-variable GMM regression models estimating the impact of gender/EDI language in job ads on labor force composition – period interactions

	% women						% racial minority	
	B (*SE*) [*p*] {*p*}	*B* (*SE*) [*p*] {*p*}	*B* (*SE*) [*p*] {*p*}	*B* (*SE*) [*p*] {*p*}	*B* (*SE*) [*p*] {*p*}	*B* (*SE*) [*p*] {*p*}	*B* (*SE*) [*p*] {*p*}	*B* (*SE*) [*p*] {*p*}
Explicit gender references (high = feminine) × period	0.026 (0.015) [0.071]							
Gendered psychological cues (high = feminine) × period		0.126 (0.065) [0.050]						
Gendered work roles (high = feminine) × period			0.033 (0.022) [0.133]					
Work-family cues (high = feminine) × period				–0.269 (0.096) [0.005]				
EDI policy (high = pro-EDI) × period					–0.043 (0.027) [0.115]		0.008 (0.006) [0.191]	
EDI culture (high = pro-EDI) × period						–0.029 (0.016) [0.060]		0.006 (0.004) [0.114]
Constant	Yes	Yes	Yes	Yes	Yes	Yes	Yes	Yes
Control variables	Yes	Yes	Yes	Yes	Yes	Yes	Yes	Yes
Instrumental variables	Yes	Yes	Yes	Yes	Yes	Yes	Yes	Yes

Note: *N* = 28,609,485 job ads for all models. SE = Standard errors clustered at the level of industry-occupation groups (189 groups). GMM = Generalized method of moments. EDI = Equality, diversity, and inclusion. LM = Lagrange multiplier. First-stage model results are not shown. Racial minority refers to all ethnic groups other than white British, white Irish, and other white groups. Control variables include the region, source and year of job ads. All instrumental variables are the same as those reported in *Supplementary Materials 7*, and they have passed all the tests reported in *Supplementary Materials 7* as valid instruments. The period dummy distinguishes between 2018–2020 and 2021–2023.

Table S16. Two-stage instrumental-variable GMM regression models estimating the *linear* impact of labor force composition on gender/EDI language in job ads – period interactions (all language scores adjusted for the year, region, and source of job ads)

	Explicit gender references	Gendered psychological cues	Gendered work roles	Work-family cues	EDI policy	EDI culture	EDI policy	EDI culture
	B (*SE*) [*p*] {*p*}	*B* (*SE*) [*p*] {*p*}	*B* (*SE*) [*p*] {*p*}	*B* (*SE*) [*p*] {*p*}	*B* (*SE*) [*p*] {*p*}	*B* (*SE*) [*p*] {*p*}	*B* (*SE*) [*p*] {*p*}	*B* (*SE*) [*p*] {*p*}
% women × period	–0.018	0.045	0.024	0.026	–0.042	0.016		
	(0.021)	(0.036)	(0.035)	(0.018)	(0.035)	(0.033)		
	[0.400]	[0.210]	[0.506]	[0.153]	[0.235]	[0.639]		
% racial minority × period							1.306	0.479
							(0.226)	(0.217)
							[< 0.001]	[0.028]
Constant	Yes	Yes	Yes	Yes	Yes	Yes	Yes	Yes
Instrumental variables	Yes	Yes	Yes	Yes	Yes	Yes	Yes	Yes

Note: *N* = 189 industry-occupation groups for all models. SE = Standard errors. GMM = Generalized method of moments. EDI = Equality, diversity, and inclusion. LM = Lagrange multiplier. First-stage model results are not shown. Racial minority refers to all ethnic groups other than white British, white Irish, and other white groups. All instrumental variables are the same as those reported in *Supplementary Materials 7*, and they have passed all the tests reported in *Supplementary Materials 7* as valid instruments. The period dummy distinguishes between 2018–2020 and 2021–2023.

Table S17. Two-stage instrumental-variable GMM regression models estimating the *non-linear* impact of labor force composition on gender/EDI language in job ads – period interactions (all language scores adjusted for the year, region, and source of job ads)

	Explicit gender references	Gendered psychological cues	Gendered work roles	Work-family cues	EDI policy	EDI culture	EDI policy	EDI culture
	B (*SE*) [*p*] {*p*}	*B* (*SE*) [*p*] {*p*}	*B* (*SE*) [*p*] {*p*}	*B* (*SE*) [*p*] {*p*}	*B* (*SE*) [*p*] {*p*}	*B* (*SE*) [*p*] {*p*}	*B* (*SE*) [*p*] {*p*}	*B* (*SE*) [*p*] {*p*}
% women × period	–0.181	–0.133	–0.115	0.049	0.321	0.112		
	(0.128)	(0.229)	(0.210)	(0.104)	(0.217)	(0.202)		
	[0.157]	[0.561]	[0.585]	0.637]	[0.140]	[0.580]		
% women (squared) × period	0.002	0.002	0.002	–0.0003	–0.004	–0.001		
	(0.001)	(0.003)	(0.002)	(0.001)	(0.002)	(0.002)		
	[0.202]	[0.425]	[0.516]	[0.774]	[0.093]	[0.624]		
% racial minority × period							–1.774	–0.609
							(0.549)	(0.435)
							[0.001]	[0.162]
% racial minority (squared) × period							0.068	0.026
							(0.015)	(0.011)
							[< 0.001]	[0.020]
Constant	Yes	Yes	Yes	Yes	Yes	Yes	Yes	Yes
Instrumental variables	Yes	Yes	Yes	Yes	Yes	Yes	Yes	Yes

Note: *N* = 189 industry-occupation groups for all models. SE = Standard errors. GMM = Generalized method of moments. EDI = Equality, diversity, and inclusion. LM = Lagrange multiplier. First-stage model results are not shown. Racial minority refers to all ethnic groups other than white British, white Irish, and other white groups. All instrumental variables are the same as those reported in *Supplementary Materials* 7, and they have passed all the tests reported in *Supplementary Materials 7* as valid instruments. The period dummy distinguishes between 2018–2020 and 2021–2023.

Table S18. Two-stage instrumental-variable GMM regression models estimating the impact of gender language in job ads on labor force racial composition

	% racial minority			
	B (*SE*) [*p*]	*B* (*SE*) [*p*]	*B* (*SE*) [*p*]	*B* (*SE*) [*p*]
Explicit gender references (high = feminine)	–0.001<br>(0.009)<br>[0.909]			
Gendered psychological cues (high = feminine)		0.007<br>(0.028)<br>[0.801]		
Gendered work roles (high = feminine)			–0.001<br>(0.012)<br>[0.939]	
Work-family cues (high = feminine)				–0.012<br>(0.033)<br>[0.705]
Constant	Yes	Yes	Yes	Yes
Control variables	Yes	Yes	Yes	Yes
Instrumental variables	Yes	Yes	Yes	Yes

Note: *N* = 28,609,485 job ads for all models. SE = Standard errors clustered at the level of industry-occupation groups (189 groups). GMM = Generalized method of moments. First-stage model results are not shown. Racial minority refers to all ethnic groups other than white British, white Irish, and other white groups. Control variables include the region, source and year of job ads. All instrumental variables are the same as those reported in *Supplementary Materials 7*, and they have passed all the tests reported in *Supplementary Materials 7* as valid instruments.

Supplementary Materials 9: Data access and replication codes

Given copyright and confidentiality issues, we are not able to share the individual job ad data. Similarly, we have used the labor force statistics curated by the Office for National Statistics in the United Kingdom (UK) through the UK Data Service after permission. While according to the data access agreement we are allowed to share the aggregate statistical findings (as reported in the Article and Supplementary Information), we do not have permission to share the original job ad or labor force data. The codes for conducting all steps of data preparation and analysis are publicly available at https://osf.io/v8b6m. To replicate our analyses, one needs to apply for access to and download the UK Quarterly Labor Force Survey data (January 2018 to June 2023; April 2001 to December 2002) via the UK Data Service (https://beta.ukdataservice.ac.uk/datacatalogue/series/series?id=2000026), as well as acquire the job ad data from Lightcast (https://lightcast.io). The codes for replicating our data preparation and analyses include the following:

- Python codes in the Jupyter notebook format for (pre)processing the job ad data and word embedding for generating gender/EDI language scores;
- Stata codes for further cleaning the job ad data and cleaning and merging in labor force statistics;
- Stata codes for conducting data analyses and robustness checks;
- R scripts for producing graphs.

Three software were used for the analyses (Python, R, and Stata), of which two were open source (Python and R). Although Stata is not an open-source software, it is a standard, state-of-the-art software for the type of econometric modeling used in our analysis. Specific software packages used include: For the job advertising preprocessing and word embedding calculation, Pythton (version 3.9.5) and specifically numpy (version 1.22.4), pandas (version 1.3.4), pytorch (version 1.13.0), nltk (version 3.6.2), genism (version 4.1.2), flair (version 0.11.3), scipy (version 1.7.1) and jupyterlab (version 3.1.11) packages were used. For the descriptive and modeling analyses presented in the main and supplementary files, Stata 18.0 MP4 was used. The instrumental-variable models were estimated using the ivreg2 package in Stata. The graphs included in the main article were produced using R (version 4.2.2) and the “ggplot2” package (version 3.4.0).

Supplementary information references

1. Organisation for Economic Co-operation and Development, *Joining forces for gender equality: What is holding us back?* (OECD Publishing, 2023).

2. Organisation for Economic Co-operation and Development, *All hands in? Making diversity work for all.* (OECD Publishing, 2020).

3. L. Quillian, et al., Do some countries discriminate more than others? Evidence from 97 field experiments of racial discrimination in hiring. *Sociol. Sci.* **6**, 467–496 (2019).

4. N. Mooten, Racism, discrimination and migrant workers in Canada: Evidence from the literature (Immigration, Refugees and Citizenship Canada Immigration, 2021).

5. Labour Market Information Council, *How representative are online job postings?* (LMI Insight Report No. 36, 2020).

6. A. P. Carnevale, T. Jayasundera, D. Repnikov, Understanding online job ads data: A technical report. (MS of PP Center on Education and the Workforce, 2014).

7. S. Ronan, *De-biasing language in job adverts.* HR Magazine UK. (2019).

8. G. Esping-Andersen, *The three worlds of welfare capitalism* (Princeton University Press, 1990).

9. D. Kamerāde, H. Richardson, Gender segregation, underemployment and subjective well-being in the UK labour market. *Hum. Relat*. **71**, 285–309 (2018).

10. I. Buchanan, A. Pratt, B. Francis-Devine, Women and the UK economy. *Res. Brief. House Commons Libr.* (2023).

11. UK Government, List of ethnic groups. Available at: https://www.ethnicity-facts-figures.service.gov.uk/style-guide/ethnic-groups/ [Accessed 1 May 2024].

12. UK Government. Ethnicity facts and figures: Employment by occupation. Available at: https://www.ethnicity-facts-figures.service.gov.uk/work-pay-and-benefits/employment/employment-by-occupation/latest/ [Accessed 1 May 2024].

13. UK Government. Outcomes in labour market for ethnic minorities by immigrant generation status (Equality Hub, Race Disparity Unit, 2023). Available at: https://www.gov.uk/government/publications/outcomes-in-labour-market-for-ethnic-minorities-by-immigrant-generation-status/outcomes-in-labour-market-for-ethnic-minorities-by-immigrant-generation-status [Accessed 1 May 2024].

14. G. Catney, A. Sabater, Ethnic minority disadvantage in the labour market: Participation, skills and geographical inequalities. Joseph Rowntree Foundation, pp. 1–96 (Full Published Report). (2015).

15. A. F. Heath, V. Di Stasio, Racial discrimination in Britain, 1969–2017: A meta-analysis of field experiments on racial discrimination in the British labour market. *Br. J. Sociol.* 70, 1774–1798 (2019).

16. McKinsey & Company, Race in the UK workplace: The intersectional experience. (McKinsey Institute for Black Economic Mobility, 2023).

17. UK Parliament, Equality Act 2010. Available at: https://www.legislation.gov.uk/ukpga/2010/15/contents [Accessed 13 March 2023].

18. A. T. Mannetje, H. Kromhout, The use of occupation and industry classifications in general population studies. *Int. J. Epidemiol.* **32**, 419–428 (2003).

19. L. Quillian, D. Pager, O. Hexel, A. H. Midtbøen, Meta-analysis of field experiments shows no change in racial discrimination in hiring over time. *Proc. Natl. Acad. Sci.* **114**, 10870–10875 (2017).

20. P. M. Blau, *Inequality and heterogeneity*. (Free Press, 1977).

21. S. L. Bem, D. J. Bem, Does sex-biased job advertising “aid and abet” sex discrimination? *J. Appl. Soc. Psychol.* **3**, 6–18 (1973).

22. I. Askehave, K. K. Zethsen, Gendered constructions of leadership in Danish job advertisements. *Gend. Work Organ.* **21**, 531–545 (2014).

23. J. Cryan, *et al.*, Detecting gender stereotypes: Lexicon vs. supervised learning methods, in *Proceedings of the 2020 CHI Conference on Human Factors in Computing Systems*, (ACM, 2020), pp. 1–11.

24. L. Wille, E. Derous, When job ads turn you down: How requirements in job ads may stop instead of attract highly qualified women. *Sex Roles* **79**, 464–475 (2018).

25. D. Gaucher, J. Friesen, A. C. Kay, Evidence that gendered wording in job advertisements exists and sustains gender inequality. *J. Pers. Soc. Psychol.* **101**, 109 (2011).

26. S. L. Bem, The measurement of psychological androgyny. *J. Consult. Clin. Psychol.* **42**, 155 (1974).

27. A. Cohen, T. Karelitz, T. Kricheli-Katz, S. Pumpian, T. Regev, Gender-neutral language and gender disparities (National Bureau of Economic Research, 2023).

28. S. Chaturvedi, K. Mahajan, Z. Siddique, Words matter: Gender, jobs and applicant behavior. *Words Matter* (2021).

29. H. Gonen, Y. Goldberg, Lipstick on a pig: Debiasing methods cover up systematic gender biases in word embeddings but do not remove them. [Preprint] (2019). Available at: http://arxiv.org/abs/1903.03862 [Accessed 1 June 2024].

30. H.-F. Hsieh, S. E. Shannon, Three approaches to qualitative content analysis. *Qual. Health Res.* **15**, 1277–1288 (2005).

31. J. Saldaña, *The coding manual for qualitative researchers* (Sage, 2021).

32. K. Krippendorff, *Content analysis: An introduction to its methodology* (Sage, 2018).

33. T. Manzini, Y. C. Lim, Y. Tsvetkov, A. W. Black, Black is to criminal as Caucasian is to police: detecting and removing multiclass bias in word embeddings. [Preprint] (2019). Available at: http://arxiv.org/abs/1904.04047 [Accessed 1 June 2024].

34. S. Guo, Difference, deficiency, and devaulation: Tracing the roots of non-recognition of foreign credentials for immigrant professionals in Canada. *Can. J. Study Adult Educ.* **22**, 37–52 (2009).

35. H. Shan, Skill as a relational construct: hiring practices from the standpoint of Chinese immigrant engineers in Canada. *Work Employ. Soc.* **27**, 915–931 (2013).

36. Y. Cha, Overwork and the persistence of gender segregation in occupations. *Gend. Soc.* **27**, 158–184 (2013).

37. E. M. Reid, O. A. O'Neill, M. Blair-Loy, Masculinity in male-dominated occupations: How teams, time, and tasks shape masculinity contests. *J. Soc. Issues* **74**, 579–606 (2018).

38. D. A. Cotter, J. M. Hermsen, R. Vanneman, *Gender inequality at work* (Russell Sage Foundation, 2004).

39. P. England, A. Levine, E. Mishel, Progress toward gender equality in the United States has slowed or stalled. *Proc. Natl. Acad. Sci.* **117**, 6990–6997 (2020).

40. C. L. Ridgeway, Framed before we know it: How gender shapes social relations. *Gend. Soc.* **23**, 145–160 (2009).

41. C. L. Ridgeway, *Framed by gender: How gender inequality persists in the modern world* (Oxford University Press, 2011).

42. J. E. Wallace, Parenthood and commitment to the legal profession: Are mothers less committed than fathers? *J. Fam. Econ. Issues* **29**, 478–495 (2008).

43. D. M. Hatmaker, Engineering Identity: Gender and Professional Identity Negotiation among Women Engineers. *Gend. Work Organ.* **20**, 382–396 (2013).

44. J. Acker, Hierarchies, jobs, bodies: A theory of gendered organizations. *Gend. Soc.* **4**, 139–158 (1990).

45. E. L. Kelly, S. K. Ammons, K. Chermack, P. Moen, Gendered Challenge, Gendered response: Confronting the ideal worker norm in a white-collar organization. *Gend. Soc.* **24**, 281–303 (2010).

46. C. West, D. H. Zimmerman, Accounting for doing gender. *Gend. Soc.* **23**, 112–122 (2009).

47. E. P. Cook, Role salience and multiple roles: A gender perspective. *Career Dev. Q.* **43**, 85–95 (1994).

48. H. Luhaorg, M. T. Zivian, Gender role conflict: The interaction of gender, gender role, and occupation. *Sex Roles* **33**, 607–620 (1995).

49. S. Sweet, *The work-family interface: An introduction* (Sage, 2013).

50. A. Killewald, A reconsideration of the fatherhood premium: marriage, coresidence, biology, and fathers' wages. *Am. Sociol. Rev.* **78**, 96–116 (2013).

51. S. Fuller, Y. Qian, Parenthood, gender, and the risks and consequences of job loss. *Soc. Forces* **100**, 1642–1670 (2021).

52. K. Weisshaar, From opt out to blocked out: The challenges for labor market re-entry after family-related employment lapses. *Am. Sociol. Rev.* **83**, 34–60 (2018).

53. H. Chung, T. van der Lippe, Flexible working, work–life balance, and gender equality: Introduction. *Soc. Indic. Res.* **151**, 365–381 (2020).

54. T. D. Allen, R. C. Johnson, K. M. Kiburz, K. M. Shockley, Work–family conflict and flexible work arrangements: Deconstructing flexibility. *Pers. Psychol.* **66**, 345–376 (2013).

55. C. Collins, *Making motherhood work: How women manage careers and caregiving* (Princeton University Press, 2019).

56. T. Koellen, Diversity management: A critical review and agenda for the future. *J. Manag. Inq.* **30**, 259–272 (2021).

57. A. M. Konrad, P. Prasad, J. Pringle, *Handbook of workplace diversity* (Sage, 2005).

58. M. D. C. Triana, P. Gu, O. Chapa, O. Richard, A. Colella, Sixty years of discrimination and diversity research in human resource management: A review with suggestions for future research directions. *Hum. Resour. Manage.* **60**, 145–204 (2021).

59. S. Yadav, U. Lenka, Diversity management: a systematic review. *Equal. Divers. Incl. Int. J.* **39**, 901–929 (2020).

60. A. M. Broadbridge, S. L. Fielden, *Research handbook of diversity and careers* (Edward Elgar, 2018).

61. E. E. Kossek, Managing work-life boundaries in the digital age. *Organ. Dyn.* **45**, 258–270 (2016).

62. M.-E. Brunet, C. Alkalay-Houlihan, A. Anderson, R. Zemel, Understanding the origins of bias in word embeddings in *Proceedings of the 36th International Conference on Machine Learning*, (PMLR, 2019), pp. 803–811.

63. O. Papakyriakopoulos, S. Hegelich, J. C. M. Serrano, F. Marco, Bias in word embeddings in *Proceedings of the 2020 Conference on Fairness, Accountability, and Transparency*, (ACM, 2020), pp. 446–457.

64. S. Hu, *et al.*, Balancing gender bias in job advertisements with text-level bias mitigation. *Front. Big Data* **5**, 805713 (2022).

65. A. Caliskan, J. J. Bryson, A. Narayanan, Semantics derived automatically from language corpora contain human-like biases. *Science* **356**, 183–186 (2017).

66. S. Vijayarani, M. J. Ilamathi, M. Nithya, Preprocessing techniques for text mining-an overview. *Int. J. Comput. Sci. Commun. Netw.* **5**, 7–16 (2015).

67. I. Burn, P. Button, L. M. Corella, D. Neumark, Does ageist language in job ads predict age discrimination in hiring? *J. Labor Econ.* **40**, 613–667 (2022).

68. E. J. Castilla, H. J. Rho, The gendering of job postings in the online recruitment process. *Manag. Sci.* **69**, 6912–6939 (2023).

69. J. Pennington, R. Socher, C. D. Manning, Glove: Global vectors for word representation in *Proceedings of the 2014 Conference on Empirical Methods in Natural Language Processing (EMNLP)*, (2014), pp. 1532–1543.

70. T. Bolukbasi, K.-W. Chang, J. Y. Zou, V. Saligrama, A. T. Kalai, Man is to computer programmer as woman is to homemaker? debiasing word embeddings. *Adv. Neural Inf. Process. Syst.* **29** (2016).

71. N. Garg, L. Schiebinger, D. Jurafsky, J. Zou, Word embeddings quantify 100 years of gender and ethnic stereotypes. *Proc. Natl. Acad. Sci.* **115**, E3635–E3644 (2018).

72. J. Dhamala, *et al.*, BOLD: Dataset and metrics for measuring biases in open-ended language generation in *Proceedings of the 2021 ACM Conference on Fairness, Accountability, and Transparency*, (ACM, 2021), pp. 862–872.

73. K. A. Bollen, Instrumental variables in sociology and the social sciences. *Annu. Rev. Sociol.* **38**, 37–72 (2012).

74. C. F. Baum, ivreg2: Stata module for extended instrumental variables/2SLS, GMM and AC/HAC, LIML and k-class regression. *Httpideas Repec Orgcbocbocodes425401 Html* (2007).

75. A. Abadie, S. Athey, G. W. Imbens, J. M. Wooldridge, When should you adjust standard errors for clustering? *Q. J. Econ.* **138**, 1–35 (2022).

76. M.L. Anderson, Multiple inference and gender differences in the effects of early intervention: A reevaluation of the Abecedarian, Perry Preschool, and Early Training Projects. *J. Am. Stat. Assoc.* **103**, 1481–1495 (2008).
